# Satisfaction and quality of life of Palestinian women with newly diagnosed breast cancer: a one-year follow-up study

**DOI:** 10.3389/fpsyg.2025.1614851

**Published:** 2025-09-22

**Authors:** Ibtisam Titi, Nuha El Sharif

**Affiliations:** ^1^Faculty of Public Health, Al Quds University, Jerusalem, Palestine; ^2^Ministry of Health, Ramallah, Palestine

**Keywords:** breast cancer, quality of life, satisfaction, newly diagnosed, Palestine

## Abstract

**Background:**

Breast cancer significantly impacts women’s quality of life (QoL), particularly in low-resource settings like Palestine. Evaluating changes in QoL and satisfaction with care is essential to improve patient-centred oncology services.

**Aim:**

This study aimed to assess satisfaction with care and quality of life (QoL) among newly diagnosed women with breast cancer in the southern West Bank, Palestine.

**Methods:**

A prospective follow-up study included 144 newly diagnosed women with breast cancer treated at governmental hospitals in the southern West Bank. Quality of life was assessed at diagnosis and one year later using the Arabic versions of the EORTC QLQ-C30 and QLQ-BR23, while satisfaction with care was measured by the EORTC IN-PATSAT32. Descriptive statistics, bivariate analysis and multiple linear regression were performed to identify factors associated with QoL after treatment.

**Results:**

The mean global health score (QLQ-C30) was 45.78, with functional and symptom scores of 43.17 and 37.19 (QLQ-BR23), respectively. Age was positively associated with body image (*B* = 22.61, 95% CI: 14.6–30.5) but negatively linked to sexual functioning (*B* = −14.44, 95% CI: −20.4-8.4), sexual enjoyment (*B* = −13.66, 95% CI: −21.1-6.1), and increased systemic side effects (*B* = 12.57, 95% CI: 6.8–18.3). Marital status improved sexual functioning (*B* = 33.89, 95% CI: 25.2–42.5) and enjoyment (*B* = 26.50, 95% CI: 15.5–37.4). Satisfaction with healthcare providers was associated with better body image (*B* = 0.34, 95% CI: 0.2–0.4) and sexual functioning (*B* = 0.19, 95% CI: 0.01–0.3). Clean, comfortable services and skilled nurses were linked to fewer therapy side effects (*B* = −0.24, 95% CI: −0.3-0.1) and less distress from symptoms like hair loss (*B* = −0.19, 95% CI: −0.35-0.0). However, Access to services was positively associated with systemic therapy side effects (*B* = 0.25, 95% CI: 0.01–0.4).

**Conclusion:**

This study demonstrated significant changes in QoL and satisfaction with care among newly diagnosed Palestinian women with breast cancer, notably exacerbated by the challenges of a country in conflict. Improving patient-centered care is essential for enhancing patient experience and treatment outcomes in Palestine’s resource-limited and frequently unstable healthcare context. Healthcare services should emphasize efficient symptom management, open communication, and enhanced psychosocial support networks for cancer patients, acknowledging the unique stressors and limited infrastructure characteristic of a conflict-affected area.

## 1 Introduction

Breast cancer represents a significant public health burden, characterized by high morbidity and mortality worldwide (Okati-Aliabad et al., 2022). Epidemiological data indicate that it remains the most prevalent malignancy among women, with over 268,000 new cases and approximately 42,000 deaths recorded in 2019 (Javan Biparva et al., 2022).

Quality of life (QoL) assessment is recognized as the most sensitive, robust indicator of breast cancer outcomes (Ai et al., 2017). Integrating QoL assessments into clinical practice enhances diagnosis accuracy, treatment efficacy, and patient monitoring by providing precise evaluations of well-being, thereby facilitating improved patient counseling (Javan Biparva et al., 2022). The World Health Organization (WHO) defines QoL as an individual’s perception of their position in life, contextualized within the cultural milieu, value systems, personal goals, expectations, standards, and concerns (World Health Organization, 1998). This multidimensional concept encompasses physical health, psychological well-being, independence, social interactions, personal beliefs, and the individual’s interaction with environmental determinants (Kotronoulas et al., 2017). This definition underscores the subjective nature of QoL, which is shaped by cultural, social, and environmental influences. According to Mokhatri-Hesari and Montazeri (2020), health-related quality of life is defined as breast cancer patients’ perception of their physical, mental, and social well-being, shaped by the stages of diagnosis, treatment, post-treatment, and survivorship, and evaluated through the use of validated measurement tools (Mokhatri-Hesari and Montazeri, 2020).

Patient satisfaction is a critical determinant of overall well-being in cancer patients (Batbaatar et al., 2015). It is defined as the degree to which an individual’s actual experiences meet or exceed their pre-existing expectations (Asadi-Lari and Gray, 2004). Several factors contribute to cancer patient satisfaction, such as access to care, the quality of the treatment environment, technical knowledge, and interpersonal interactions with healthcare providers. Moreover, satisfaction is influenced by the clarity and sufficiency of information delivered by medical professionals, their sensitivity to the patient’s health and treatment-related challenges, their comprehension of emotional requirements, and their capacity for effective communication (Batbaatar et al., 2017). Patient satisfaction is widely seen as an essential indicator of care quality, including the comprehensive healthcare experience (Gomez-Cano et al., 2020).

As a critical indicator of healthcare quality, patient satisfaction facilitates the identification of areas for enhancement, enabling healthcare providers to refine service delivery and policymakers to formulate strategies that more effectively address patient needs (Batbaatar et al., 2017; Kleeberg et al., 2005). Furthermore, patient satisfaction plays a pivotal role in breast cancer interventions, particularly in surgical outcomes, by influencing treatment adherence (Pintault et al., 2023). The association between QoL and satisfaction with healthcare services is well-established in oncology patients (Asadi-Lari and Gray, 2004; Djambazov and Giammanco, 2019). Comprehensive assessment of QoL and satisfaction is significant for identifying breast cancer patients requiring tailored support, as these domains are interconnected and provide a holistic understanding of patients’ experiences (Brédart et al., 2013). Patients exhibiting higher satisfaction levels are more likely to demonstrate sustained engagement with healthcare services and adherence to treatment protocols (Abate et al., 2020).

In the field of breast cancer, several studies have shown that patient satisfaction is a critical factor influencing psychological adjustment, body image, and emotional well-being (Baumbach et al., 2023; Saiga et al., 2023). Therefore, examining the correlation between satisfaction and quality of life is vital for understanding the long-term experiences of women receiving cancer treatment (Shiraishi et al., 2022). In a longitudinal study by Krzos et al. (2019), QoL and satisfaction with breast-conserving surgery outcomes were assessed. The findings revealed that patients’ satisfaction with breast appearance improved significantly three months after surgery and was positively correlated with higher psychosocial well-being. Physical well-being decreased, but psychosocial QoL increased, especially twelve months following surgery. This study emphasized that higher satisfaction with surgical outcomes is significantly associated with improved perceptions of psychosocial quality of life (Krzos et al., 2019).

In the Palestinian context, breast cancer maintains its status as the most common neoplastic disease, exhibiting an incidence rate of 18.6 cases per 100,000 individuals within the West Bank in 2023 (MOH, 2024). Despite this high prevalence, to our knowledge, no studies in Palestine have specifically examined the interrelationship between patient satisfaction and quality of life (QoL) among newly diagnosed women with breast cancer. Furthermore, most existing evidence originates from high-resource settings, leaving a critical gap in knowledge from low-resource regions such as Palestine, where access to care, psychosocial support, and treatment continuity may be compromised. Therefore, this study aimed to assess satisfaction with care and QoL among newly diagnosed women with breast cancer in the southern West Bank, Palestine.

## 2 Materials and methods

### 2.1 Study design

A prospective follow-up study design was used, with data collected at two time points: diagnosis (T1) and one year later (T2).

### 2.2 Study context and population

The Palestinian health system is comparable to those of other low-to-middle-income countries. The Palestinian Ministry of Health (MoH) serves as the primary healthcare provider in collaboration with NGOs and the private sector to promote cancer prevention and control (Jubran et al., 2022). In the southern West Bank, cancer patients are referred to two governmental hospitals—one in the Bethlehem governorate and the other in the Hebron governorate—for diagnosis, treatment, and inpatient care (Mitwalli et al., 2023). In alignment with the Division of Cancer Control and Population Sciences (DCCPS) definition, newly diagnosed cancer patients are individuals with a first-time cancer diagnosis who are currently undergoing treatment, have completed treatment, or are managing progressive symptoms (DCCPS, 2024).

### 2.3 Participant recruitment

This study tracked women undergoing treatment at government hospitals in the southern West Bank after receiving a recent breast cancer diagnosis. Participants who were diagnosed and formally registered between August 2023 and July 2024 were eligible. Each participant was followed prospectively for one year after her diagnosis to evaluate changes in quality of life and satisfaction with care. Data collection was conducted between November 2024 and January 2025 and included only those participants who had completed the full one-year follow-up period.

A total of 2,502 invasive breast cancer cases were registered in the West Bank between 2017 and 2021. Therefore, approximately 500 new women are diagnosed yearly. The Ministry of Health annual report (2024) indicated that 546 new cases of breast cancer were diagnosed in the West Bank in 2023, with 161 of these cases occurring in the Bethlehem and Hebron governorates (MOH, 2024). This study employed a purposive sampling strategy to recruit newly diagnosed women with breast cancer from two governmental hospitals in the southern West Bank. A total of 362 patients in a previous study conducted by the research team, among whom 147 met the inclusion criteria (TitiI, 2024). During the final stage of data collection, three patients passed away; therefore, the final study sample comprised 144 newly diagnosed women.

### 2.4 Outcomes of interest

The study primarily measured two outcomes. The QoL was measured by EORTC QLQ-C30 (T1) at diagnosis and EORTC QLQ-BR23 (T2) after one year. The second outcome was satisfaction of care using EORTC IN-PATSAT32 (T2).

### 2.5 Study tools

Three standardized questionnaires were used. We used the European Organization for Research and Treatment of Cancer QoL (EORTC) QLQ-C30 and the QoL EORTC BR23 Questionnaires to assess patient QoL. Also, for patient satisfaction with care, we used the Arabic version of the Cancer In-Patient Satisfaction Questionnaire (EORTC IN-PATSAT32). All data were collected through face-to-face interviews during both the baseline and 1-year follow-up phases at governmental hospitals in the southern West Bank. At baseline, the EORTC QLQ-C30 was administered, while at the 1-year follow-up, participants completed the EORTC QLQ-BR23 and EORTC IN-PATSAT32 questionnaires. Additionally, we added questions on patients’ demographic data, disease-related information, socioeconomic status, age, cultural norms, educational background, beliefs, personality traits, cancer stage, area of residence, and levels of social and familial support.

#### 2.5.1 Quality of life at diagnosis

**The EORTC QLQ-C30 questionnaire** includes 30 items divided into nine scales: five functional scales (physical, role, cognitive, emotional, and social), three symptom scales (fatigue, pain, and nausea/vomiting), and one global health and quality-of-life scale (Bishop et al., 2015). Each scale is scored from 0 to 100, with higher scores indicating better QoL on the functional and global health scales but greater symptom severity on the symptom scales (Tan et al., 2014). The Arabic version of the EORTC QLQ-C30 used in this study has been validated for breast cancer patients (Jassim and Alansari, 2020). The questionnaire demonstrated high reliability, with an overall Cronbach’s alpha of 0.91.

#### 2.5.2 Quality of life after treatment

**The EORTC QLQ-BR23** is a 23-item breast cancer-specific questionnaire developed by the European Organization for Research and Treatment of Cancer (EORTC) to assess QoL in breast cancer patients, particularly across different stages of the disease and treatment modalities (Sprangers et al., 1996). The questionnaire has two main dimensions: a functional scale (body image, sexual function, sexual enjoyment, and future perspective) and a symptom scale (treatment side effects, breast and arm symptoms, and hair loss distress). Items are rated from 1 (not at all) to 4 (very much), with higher functional scores indicating better QoL and higher symptom scores reflecting greater distress (Kostic et al., 2020). The scoring method is similar to that of the EORTC QLQ-C30 questionnaire, providing a consistent approach for measuring QoL in cancer patients (Vieira et al., 2023). The questionnaire demonstrated high reliability, with an overall Cronbach’s alpha of 0.79.

#### 2.5.3 Cancer in-patient satisfaction questionnaire

**The EORTC IN-PATSAT32** is a multidimensional, 32-item questionnaire developed by the European Organisation for Research and Treatment of Cancer (EORTC) to assess cancer patients’ satisfaction with various aspects of oncology care, including the quality of medical services, hospital environment, and overall service organisation (Brédart et al., 2005). It was validated through a multi-centre study evaluating patients’ perceptions of doctors, nurses, hospital staff, services, and the hospital environment (Obtel et al., 2017). The questionnaire consists of 11 multi-item scales and 3 single-item scales, measuring technical and interpersonal skills, information provision, and availability of doctors and nurses. Additionally, it assesses hospital personnel’s communication, waiting times, hospital accessibility, information exchange, and hospital cleanliness and comfort. Each item is rated on a 5-point Likert scale (1 = poor to 5 = excellent), with higher scores indicating greater patient satisfaction (Abdelsalam and Bayomi, 2022). The questionnaire demonstrated high reliability, with an overall Cronbach’s alpha of 0.98.

### 2.6 Statistical analysis

Data analysis was conducted using IBM SPSS Statistics (version 25). The statistical analysis of the EORTC QLQ-C30 begins with calculating raw scores for each scale by averaging the responses to the component items. For the functional scales, the raw score (RS) is transformed to a 0–100 scale using the formula S = [(RS – 1)/3] × 100, so that higher scores indicate better functioning. In contrast, for the symptom scales, the transformation is inverted with S = [1 – (RS – 1)/3] × 100, ensuring that higher scores reflect more severe symptoms. For the global health status, which is measured on a 7-point scale, the formula S = [(RS – 1)/6] × 100 is used. The scoring approach for the QLQ-BR23 is identical in principle to that for the function and symptom scales/single items of the QLQ-C30.

Spearman’s rank correlation test was used to examine the strength and direction of associations between QLQ-BR23 item scores and all other scales, with statistical significance defined at *p* < 0.05. The normality of all data was assessed using the Kolmogorov–Smirnov test, with a significance level set at *p* < 0.05. The bivariate analyses examined relationships between sociodemographic factors, clinical variables, and social support with QLQ-BR23 using the Mann–Whitney U and Kruskal–Wallis tests. We applied a multiple linear regression to examine the factors influencing QLQ-BR23 subscales as dependent variables. Independent variables included QLQ-C30 subscales, sociodemographic variables, medical variables, social support, and satisfaction scale. Separate models were run for each QLQ-BR23 domain. Standardization was also performed for the EORTC IN-PATSAT32 scale to allow scores to be expressed on a common scale, thus facilitating comparison. Following standardization, descriptive statistics (means, standard deviations, ranges) were computed to summarize the data. Reliability analyses, such as Cronbach’s alpha, were conducted to assess the internal consistency of multi-item scales.

### 2.7 Ethical considerations

We obtained approval from the Al-Quds University Research Ethical Committee (reference number REF. 10/24) and approval from the Health Education and Scientific Research Department of the Ministry of Health (reference number 162/475/2024). In addition, all participants were informed about the study objectives and signed an informed consent form emphasizing participant confidentiality and their right to withdraw from the study at any time without impacting their clinical treatments.

## 3 Results

### 3.1 Socio-demographic and clinical characteristics of the participants

Table 1 displays the demographic characteristics of the participants. This study included 144 newly diagnosed females with breast cancer, predominantly from Hebron (70.8%), with a majority (91.7%) identifying as Muslim. Participants’ residences varied, with 62.5% residing in villages, 29.9% in cities, and 7.6% in refugee camps. Nearly half of the participants were aged ≤45 years (52.1%), and the majority were married (86.1%). In terms of education, 46.5% held a college degree, and most (71.5%) were housewives. The majority of families were large (64.6%), and 68.1% had ≤5 children. Income levels varied, with 36.1% earning <570 US dollars monthly (see Table 1).

**Table 1:** Socio-demographic and economic characteristics of participants (*N* = 144).

Variables	No.	%
Patient governorate		
Hebron	102	70.8%
Bethlehem	42	29.2%
Patient religion		
Muslim	132	91.7%
Christian	12	8.3%
Place of residence		
Refugee camps	11	7.6%
Village	90	62.5%
City	43	29.9%
Age in years		
≤45	75	52.1%
>45	69	47.9%
Marital status		
Unmarried	20	13.9%
Married	124	86.1%
Education		
Primary and less	19	13.2%
Secondary	58	40.3%
College/University	67	46.5%
Working status		
Employee	41	28.5%
Housewife	103	71.5%
Number of children		
≤5 persons	98	68.1%
>5 persons	46	31.9%
Family size (extended family)		
≤5 persons	51	35.4%
>5 persons	93	64.6%
Monthly salary (US $)		
Low (< 570)	52	36.1%
Moderate (570–1,140)	60	41.7%
High (> 1,140)	32	22.2%

### 3.2 Clinical characteristics of the participants

Table 2 presents the participants’ clinical characteristics. At diagnosis, 45.1% were in Stage 3. Regarding treatment, 97.2% received chemotherapy, 72.9% radiotherapy, and all underwent surgery (40.3% complete mastectomy, 59.7% partial mastectomy). Chronic diseases were present in 32.6, and 52.1% had a family history of cancer, with 27.8% having a relative with breast cancer. Social support varied, with 86.1% receiving family support, but 69.4% lacked husband support, and only 18.1% had other forms of support (see Table 2).

**Table 2:** Clinical characteristics and support for participants (*N* = 144).

Variables	No.	%
Stage of disease at diagnosis		
Stage 1	11	7.6%
Stage 2	58	40.3%
Stage 3	65	45.1%
Stage 4	10	6.9%
Type of treatment		
Chemotherapy	347	97.2%
Radiotherapy	246	72.9%
Hormonal therapy	194	85.4%
Surgical therapy	295	100%
Biological treatment	136	51.4%
Surgical intervention		
Total mastectomy	58	40.3%
Partial mastectomy	86	59.7%
Chronic diseases other than cancer		
Yes	47	32.6%
No	97	67.4%
Taking pain medication		
Yes	85	59.0%
No	59	41.0%
History of relative cancer		
Breast cancer	40	27.8%
Other cancers	35	24.3%
No cancer history	69	47.9%
Family support		
Yes	124	86.1%
No	20	13.9%
Husband/partner support		
Yes	44	30.6%
No	100	69.4%
Other sources of support*		
Yes	26	18.1%
No	118	81.9%

*Other source of support such as friends, colleague sand health team.

### 3.3 Quality of life scale analysis

#### 3.3.1 Quality of life at diagnosis (EORTC QLQ-C30 scale)

Data from the EORTC QLQ-C30 scale revealed variations across functional and symptom domains at the time of diagnosis. Among the functional domains, physical functioning and cognitive scale exhibited the highest mean score of 67.13 (SD = 23.75), indicating a relatively higher level of physical capacity.

However, role functioning and emotional functioning were reported at moderate levels. In contrast, social functioning had the lowest mean score of 34.49 (SD = 24.43), indicating significant challenges in this domain. In terms of symptom domains, fatigue, insomnia, and dyspnea were reported at moderate levels. Other symptoms, such as diarrhea and nausea/vomiting, had relatively low mean scores, 19.10 (SD = 26.52), suggesting that these symptoms were either less frequent or less severe. The mean score for global health status was 45.78 (SD = 17.68), reflecting a moderate overall QoL among the newly diagnosed breast cancer patients at the beginning of their treatment journey (see Table 3).

**Table 3:** Quality of life at diagnoses, QoL after one-year treatment and patients satisfaction means scales.

Variables	Mean ± SD
QoL before treatment	
EORTC QLQ-C30 functional scale	
Physical functioning	67.13 ± 23.75
Cognitive functioning	62.85 ± 30.07
Role functioning	58.56 ± 25.24
Emotional functioning	49.19 ± 25.91
Social functioning	34.49 ± 24.43
EORTC QLQ-C30 symptoms scale	
Fatigue	44.68 ± 21.21
Insomnia	42.59 ± 36.86
Dyspnea	40.05 ± 36.47
Pain	39.81 ± 22.40
Constipation	24.07 ± 34.01
Appetite loss	23.38 ± 29.52
Diarrhea	21.30 ± 31.44
Nausea vomiting	19.10 ± 26.52
Financial difficulties	37.50 ± 33.65
Global health	45.78 ± 17.68
QoL one year after treatment	
EORTC QLQ-C23 functional scale	
Body image	43.17 ± 30.67
Sexual functioning	34.49 ± 24.43
Sexual enjoyment	34.72 ± 28.39
Future perspective	23.38 ± 29.52
EORTC QLQ-C23 symptoms scale	
Arm symptoms	37.19 ± 28.41
Breast symptom	33.33 ± 25.24
Systemic therapy side effects	26.22 ± 20.34
Upset by hair loss	14.81 ± 28.37
Patient satisfaction	
EORTC IN-PATSAT32 - Doctors	
Technical skills	68.13 ± 25.18
Information provision	45.99 ± 26.14
Availability	42.36 ± 27.59
Interpersonal skills	40.20 ± 27.47
EORTC IN-PATSAT32 - Nurses	
Technical skills	63.27 ± 27.00
Interpersonal skills	53.09 ± 30.55
Information provision	42.44 ± 30.78
Availability	39.47 ± 30.71
EORTC IN-PATSAT32 - Others	
Exchange information	53.94 ± 25.55
General satisfaction	45.60 ± 28.90
Comfort/cleanliness	44.44 ± 31.78
Kindness, helpfulness, info giving	43.90 ± 23.41
Access	35.42 ± 26.21
Waiting time	33.68 ± 23.11

#### 3.3.2 Quality of life one year after treatment (QLQ-BR23)

The QLQ-BR23 functional domain data indicated moderate concerns regarding body image, with mean score of 43.17 (SD = 30.67) one year after treatment. However, participants reported low scores for sexual functioning, sexual enjoyment, with the lowest score observed for future perspective (mean = 23.38, SD = 29.52), suggesting significant uncertainty about the future. For the symptoms domain, systemic therapy side effects, breast symptoms, and arm symptoms were relatively low. Notably, the mean score for being upset by hair loss was the lowest, at 14.81 (SD = 28.37) (see Table 3).

#### 3.3.3 Patient satisfaction (EORTC IN-PATSAT32)

The EORTC IN-PATSAT32 analysis revealed the highest level of satisfaction with doctors’ technical skills, with a mean of 68.13 (SD = 25.18). However, satisfaction scores with doctors’ interpersonal skills (mean = 40.20, SD = 27.47) and information provision (mean = 45.99, SD = 26.14) were moderate. Nurse technical skills were highly rated at 63.27 (SD = 27.00), while nurses’ interpersonal skills and information provision were moderately rated. However, nurses’ availability showed the lowest rating score (mean = 39.47, SD = 30.71). Additionally, moderate satisfaction scores were reported for information exchange, general satisfaction, comfort and cleanliness, and nurses’ kindness and helpfulness. In contrast, the lowest satisfaction scores were recorded for waiting time (mean = 33.68, SD = 23.11) and access to care (mean = 35.42, SD = 26.21) (see Table 3).

### 3.4 Bivariate analysis of the EORTC QLQ-C23 scales

Table 4 presents the associations between functional aspects of QoL, as measured by the EORTC QLQ-C23 questionnaire, and various sociodemographic, clinical, and social support factors among breast cancer patients. The findings show that age was significantly associated with multiple functional outcomes. Younger patients (age ≤45 years) reported poorer body image (*p* < 0.001) but higher sexual functioning (*p* < 0.001) and sexual enjoyment (*p* = 0.001) compared to older patients (age >45 years). Unmarried women had poorer body image, lower sexual functioning, and lower sexual enjoyment (*p* < 0.05) than married women. Patients with lower education levels (primary or less) exhibited poorer body image and sexual functioning and reduced sexual enjoyment (*p* < 0.05). In contrast, patients with higher education (university level) scored better in these domains (*p* < 0.05). Employed women reported significantly better sexual functioning (*p* < 0.05) compared to housewives. Moreover, patients diagnosed at Stage 4 had significantly poorer future perspectives (*p* < 0.05). The presence of a chronic disease was associated with worse body image, lower sexual functioning, and reduced sexual enjoyment (*p* < 0.001). Similarly, patients using pain medications had significantly lower sexual functioning, sexual enjoyment, and future perspectives (*p* < 0.05). Patients not receiving chemotherapy had significantly better future perspectives (*p* < 0.05). Biological treatment was linked to better sexual functioning (*p* = 0.009) and sexual enjoyment (*p* < 0.05). However, complete mastectomy was associated with poorer body image and lower sexual enjoyment (*p* < 0.05) compared to partial mastectomy. In addition, women without a husband’s support had significantly lower sexual functioning and sexual enjoyment (*p* < 0.001), while family support did not affect these domains (see Table 4).

**Table 4:** QLQ-C23 Functional scales mean rank and its association with the study’s socio-demographic, clinical, and social support variables (*N* = 144).

Variables	Body image	Sexual functioning	Sexual enjoyment	Future perspective	Systemic therapy side effects	Breast symptom	Arm symptoms	Upset by hair loss
Residence								
Refugee camp	54.77	68.73	72.14	73.59	93.73	94.05	88.59	69.32
Village	75.24	70.36	69.85	67.49	71.92	69.69	69.26	73.59
City	71.29	77.95	78.14	82.71	68.29	72.87	75.16	71.03
***P***	0.29	0.57	0.52	0.09	0.19	0.18	0.30	0.85
Age in years								
≤45	54.09	86.03	86.03	86.03	60.13	68.67	68.93	72.74
>45	92.51	57.80	57.80	57.80	85.94	76.67	76.38	72.24
***P***	**< 0.001**	**< 0.001**	**0.001**	0.71	**< 0.001**	0.25	0.28	0.92
Marital status								
Unmarried	87.70	20.00	26.98	77.33	81.10	73.08	71.78	79.40
Married	70.05	80.97	79.84	71.72	71.11	72.41	72.62	71.39
***P***	**0.04**	**< 0.001**	**< 0.001**	0.54	0.32	0.95	0.93	0.29
Education								
Primary and less	99.95	47.50	48.87	68.45	94.92	93.53	94.13	76.71
Secondary	71.96	72.73	72.97	73.99	71.45	68.28	72.62	66.76
Colleague/University	65.19	79.39	78.80	72.36	67.05	70.19	66.26	76.28
***P***	**0.01**	**0.01**	**0.01**	0.85	**0.04**	0.06	**0.03**	0.20
Working status								
Employee	74.06	85.50	81.85	81.85	68.15	69.56	67.07	69.26
Housewife	71.88	67.33	68.78	68.78	74.23	73.67	74.66	73.79
***P***	0.77	**0.01**	0.07	0.64	0.42	0.59	0.32	0.44
Number of children								
≤5 persons	69.89	73.22	74.48	71.59	71.05	69.23	69.46	71.83
>5 persons	78.07	70.97	68.27	74.43	75.59	79.47	78.98	73.93
***P***	0.27	0.75	0.37	0.67	0.54	0.17	0.19	0.71
Family size								
≤5 persons	81.88	67.50	66.35	75.60	69.23	65.00	70.11	70.07
>5 persons	67.35	75.24	75.87	70.80	74.30	76.61	73.81	73.83
***P***	**0.04**	0.27	0.16	0.46	0.48	0.10	0.607	0.49
Income (US $)								
<570	69.07	70.00	66.60	73.77	72.84	70.63	67.02	71.38
570–1,140	72.64	72.13	73.53	72.34	73.18	68.76	71.81	75.45
>1,140	77.81	77.27	80.17	70.73	70.67	82.55	82.70	68.78
***P***	0.64	0.72	0.29	0.93	0.96	0.29	0.23	0.59
Stage at diagnosis								
Stage 1	71.82	81.86	74.36	78.50	76.59	55.91	60.77	72.50
Stage 2	78.36	69.34	72.40	72.23	74.73	79.02	80.84	77.55
Stage 3	70.45	75.25	73.82	66.68	71.09	69.20	67.26	67.32
Stage 4	52.60	62.60	62.50	80.00	64.20	74.40	71.10	76.90
***P***	0.31	0.62	0.86	**0.04**	0.86	0.30	0.23	0.33
Chemotherapy								
Yes	72.95	72.09	72.51	71.57	73.14	72.74	72.42	72.58
No	56.88	86.88	72.25	105.13	50.25	64.00	75.38	69.75
***P***	0.45	0.48	0.99	0.08	0.28	0.68	0.89	0.86
Radiotherapy								
Yes	74.43	74.00	72.95	70.42	74.45	73.75	77.07	71.73
No	67.31	68.47	71.28	78.10	67.24	69.14	60.21	74.58
***P***	0.36	0.47	0.82	0.27	0.35	0.55	**0.03**	0.63
Hormonal therapy								
Yes	71.15	74.22	72.95	70.03	70.94	72.20	73.04	68.91
No	80.43	62.40	69.86	86.98	81.64	74.26	69.33	93.55
***P***	0.34	0.22	0.74	0.06	0.28	0.83	0.70	**0.001**
Biological treatment								
Yes	65.56	81.22	79.51	79.51	67.41	67.61	67.34	71.39
No	79.84	63.28	65.09	65.09	77.88	77.67	77.96	73.67
***P***	**0.04**	**0.01**	**0.03**	**0.02**	0.13	0.15	0.12	0.66
Surgical intervention								
Complete mastectomy	60.28	64.97	64.57	74.47	75.59	65.82	57.09	69.57
Partial mastectomy	80.74	77.58	77.85	71.17	70.41	77.01	82.90	74.48
***P***	**< 0.001**	0.07	**0.04**	0.61	0.46	0.11	**< 0.001**	0.36
Chronic disease								
Yes	93.36	52.56	60.98	78.34	84.27	77.33	79.01	65.01
No	62.39	82.16	78.08	69.67	66.80	70.16	69.35	76.13
***P***	**< 0.001**	**< 0.001**	**0.01**	0.19	**0.01**	0.33	0.19	**0.04**
Pain medication								
Yes	77.71	64.71	66.22	78.19	77.38	76.12	72.62	74.54
No	65.00	83.73	81.55	64.31	65.47	67.29	72.32	69.57
***P***	0.07	**0.01**	**0.02**	**0.03**	0.09	0.21	0.97	0.35
Relatives cancer								
Breast Cancer	71.65	77.78	76.55	78.69	73.19	66.41	72.35	83.85
Other Cancers	70.16	73.73	72.24	70.94	76.57	76.96	75.41	70.79
No history of cancer	74.18	68.82	70.28	69.70	70.04	73.77	71.11	66.79
***P***	0.89	0.53	0.72	0.47	0.75	0.51	0.88	**0.02**
Family support								
Yes	72.74	74.11	74.58	72.22	72.71	75.92	73.92	71.70
No	71.00	62.53	59.63	74.25	71.20	51.33	63.68	77.45
***P***	0.86	0.24	0.11	0.82	0.88	**0.01**	0.30	0.45
Husband support								
Yes	0.153	89.88	94.48	67.50	65.55	72.02	80.31	70.11
No	0.153	64.86	62.83	74.70	75.56	72.71	69.07	73.55
***P***	0.07	**0.001**	**< 0.001**	0.29	0.18	0.93	0.13	0.55
Other support								
Yes	83.31	68.62	65.02	67.29	69.13	64.85	66.87	70.31
No	70.12	73.36	74.15	73.65	73.24	74.19	73.74	72.98
***P***	0.14	0.59	0.28	0.44	0.65	0.30	0.44	0.70

### 3.5 Spearman correlations between QLQ-BR23 scales, patient satisfaction, and QoL at diagnosis

#### 3.5.1 Quality of life

Most QoL at diagnosis scale was weakly correlated with QoL after one year of treatment, either positively or negatively corrected (*r* < 0.40). However, body image was negatively corrected with physical (*r* = −0.26), role (*r* = −0.26), and social functioning (*r* = −0.26) (*p* < 0.05), indicating its effect on daily activities. Sexual functioning was weakly positively correlated with physical (*r* = 0.290), cognitive (*r* = 0.25), and social functioning (*r* = 0.28), while was weakly negatively correlated with fatigue (*r* = −0.24), pain (*r* = −0.25), and dyspnea (*r* = −0.248) (*p* < 0.05). Future perspective had a moderately positive correlation with fatigue (*r* = 0.42), pain (*r* = 0.461), insomnia (*r* = 0.47), nausea/vomiting (*r* = 0.57), and appetite loss (*r* = 1.000), showing its strong association with symptom burden. In addition, systemic therapy side effects had weak negative correlations with physical (*r* = −0.21), role (*r* = −0.18), and social functioning (*r* = −0.32) (*p* < 0.05), suggesting an impact on overall well-being (see Table 5).

**Table 5:** Spearman’s correlation between QLQ-BR23, QoL (EORTC QLQ-C30), and satisfaction of care scales.

Variables	Body image	Sexual functioning	Sexual enjoyment	Future perspective	Systemic therapy side effect	Breast symptom	Arm symptoms	Upset by hair loss
**EORTC QLQ-C30 scales**								
Physical functioning	−0.26**	0.29**	0.19*	−0.24**	−0.21*	−0.19*	−0.10	0.03
Role functioning	−0.26**	0.12	0.05	−0.35**	−0.18*	−0.21*	−0.09	0.13
Emotional functioning	0.13	0.02	−0.03	−0.37**	−0.02	−0.06	−0.08	−0.04
Cognitive functioning	−0.11	0.25**	0.23**	−0.23**	−0.17*	−0.22**	−0.12	0.04
Social functioning	−0.26**	0.28**	0.24**	−0.08	−0.32**	−0.19*	−0.14	−0.11
Fatigue	0.07	−0.24**	−0.15	0.42**	0.19*	0.26**	0.21**	−0.06
Nausea/Vomiting	−0.10	0.03	0.03	0.57**	−0.15	−0.020	−0.04	0.03
Pain- Linear	0.18*	−0.25**	−0.15	0.46**	0.20*	0.26**	0.22**	−0.07
Dyspnea	0.22**	−0.24**	−0.23**	0.24**	0.18*	0.13	0.07	−0.04
Insomnia	−0.06	−0.12	−0.09	0.47**	0.10	0.03	−0.01	0.05
Appetite loss	−0.03	−0.08	−0.06	1.00**	0.00	−0.07	−0.05	0.05
Constipation	−0.07	−0.03	0.02	0.16*	−0.01	0.06	−0.06	−0.10
Diarrhea	−0.03	0.02	0.02	0.22**	0.04	0.06	−0.07	−0.06
Financial	0.11	−0.14	−0.13	0.17*	0.22**	0.12	0.14	−0.09
Global health	−0.03	0.28**	0.24**	−0.17*	−0.34**	−0.21*	−0.22**	0.01
EORTCIN-PATSAT32- Doctors								
Technical skills	0.28**	−0.03	−0.003	0.04	−0.03	−0.06	−0.05	−0.14
Interpersonal skills	0.37**	−0.17*	−0.17*	−0.03	0.02	−0.07	−0.02	−0.12
Information provision	0.24**	−0.10	−0.01	0.02	0.03	−0.01	−0.01	−0.09
Availability	0.32**	−0.15	−0.20*	0.04	0.02	−0.01	−0.06	−0.06
EORTCIN-PATSAT32- Nurse								
Technical skills	0.26**	0.01	0.04	−0.09	−0.05	−0.05	−0.06	−0.19*
Interpersonal skills	0.27**	−0.09	−0.07	−0.16	0.03	−0.04	−0.01	−0.13
Information provision	0.21*	−0.10	−0.10	−0.18*	0.04	0.02	0.01	−0.12
Availability	0.35**	−0.19*	−0.17*	−0.06	0.08	0.06	0.04	−0.13
EORTCIN-PATSAT32- Others								
Exchange information	0.21**	−0.01	0.01	−0.03	0.09	−0.01	−0.01	−0.07
Kindness, helpfulness, information giving	0.21**	−0.01	0.02	−0.07	0.05	−0.11	−0.04	−0.07
Waiting Time	0.16*	0.03	0.08	−0.12	0.02	−0.05	−0.03	−0.06
Access	0.17*	0.05	0.114	−0.07	0.05	−0.03	−0.01	−0.05
Comfort/cleanliness	0.03	0.06	0.12	0.01	−0.16*	−0.22**	−0.14	−0.07
General satisfaction	0.28**	0.01	−0.01	−0.09	−0.04	−0.12	−0.06	−0.08

Bold are significant values (2-tailed) *p < 0.05; **p < 0.01.

#### 3.5.2 Patient satisfaction

The correlations between patient satisfaction and the QoL after one year of treatment were weak. Body image showed positive correlations with doctor technical skills (*r* = 0.27), doctor interpersonal skills (*r* = 0.37), doctor availability (*r* = 0.32), and nurse technical skills (*r* = 0.26) (p -value< 0.05), indicating that better body image is linked to higher satisfaction with healthcare providers. It was also positively correlated with general satisfaction (*r* = 0.28). Sexual functioning and enjoyment had weak negative correlations with doctor and nurse interpersonal skills and availability (*p* < 0.05). Future perspective showed weak negative correlations with nurse interpersonal skills (*r* = −0.16) and nurse information provision (*r* = −0.17) (*p* < 0.05) (see Table 5).

### 3.6 Multiple linear regression results

In Table 6, we present the multiple linear regression analysis of the study’s various factors associated with QoL after one year of treatment (QLQ-BR23) among newly diagnosed Palestinian women with breast cancer.

**Table 6:** Multiple linear regression of Quality-of-life after treatment (QLQ-BR23) with QoL at diagnosis (QLQ-C30), sociodemographic, medical, social support variables, and patient’s satisfaction.

Variables	B	Std. error	95% CI (Lower, Upper)	*R* ^2^
Body image				
Age	22.61	4.02	(14.6, 30.5)	0.21
Husband support	11.80	4.15	(3.5, 20.0)	0.47
Surgical intervention	−15.02	5.07	(−25.1, −4.1)	0.30
Biological treatment	11.75	3.85	(4.1, 19.3)	0.40
**QoL**-Role functioning	−0.25	0.07	(−0.4, −0.0)	0.44
**Satisfaction-**Doc. interpersonal skills	0.34	0.07	(0.2, 0.4)	0.37
Sexual functioning				
Age	−14.44	3.04	(−20.4, −8.4)	0.34
Marital status	33.89	4.37	(25.2, 42.5)	0.23
Biological treatment	−9.51	3.05	(−15.5, −3.4)	0.43
**QoL-**Global health	0.19	0.09	(0.003,0.394)	0.47
**QoL-**Fatigue	−8.29	3.41	(−15.0, −1.5)	0.39
**Satisfaction-**Doc. interpersonal skills	0.19	0.09	(0.01, 0.3)	0.0.46
Sexual enjoyment				
Age	−13.66	3.78	(−21.1, −6.1)	0.25
Marital status	26.50	5.52	(15.5, 37.4)	0.17
Husband_ support	−13.92	4.15	(−22.1, −5.7)	0.31
**QoL-**Cognitive Functioning	0.13	0.06	(0.00, 0.3)	0.45
**QoL-**Diarrhea	0.12	0.06	(0.01, 0.2)	0.43
**QoL-**Global health	0.29	0.11	(0.06, 0.5)	0.34
**Satisfaction-**Doctor Availability	−0.30	0.08	(−0.46, 0.1)	0.36
**Satisfaction-**Exchange information	0.26	0.09	(0.1, 0.4)	0.38
Future perspective				
Pain	0.32	0.10	(0.1, 0.5)	0.44
Biological treatment	−11.43	3.53	(−18.4, −4.4)	0.47
Chronic disease	−10.14	3.77	(−17.6, −2.6)	0.49
Chemotherapy treatment	23.46	10.80	(2.0, 44.80)	0.53
Husband support	7.76	3.77	(0.3, 15.2)	0.54
**QoL-**Nausea vomiting	0.47	0.07	(0.3, 0.6)	0.32
**QoL-**Insomnia	0.21	0.05	(0.1, 0.3)	0.41
**QoL-**Financial difficulties	−0.12	0.06	(−0.2, −0.01)	0.51
Systemic therapy side effect				
Age	12.57	2.89	(6.8, 18.3)	0.22
**QoL-**Global health	−0.41	0.08	(−0.5, −0.2)	0.11
**Satisfaction-**Comfort/cleanliness	−0.24	0.06	(−0.3, −0.1)	0.25
**Satisfaction-**Access	0.25	0.07	(0.01, 0.4)	0.30
Breast symptom				
Family support	−18.39	5.57	(−29.4, −7.3)	0.16
Surgical intervention	9.50	3.93	(1.7, 17.2)	0.20
**QoL-**Pain	0.30	0.08	(0.1, 0.40)	0.11
**Satisfaction-**Comfort/cleanliness	−0.22	0.06	(−0.3, −0.1)	0.05
Arm symptoms				
Surgical intervention	22.26	4.48	(13.4, 31.1)	0.08
Biological treatment	10.69	4.39	(2.0, 19.3)	0.20
Education level	−6.32	3.17	(−12.5, −0.0)	0.22
**QoL-**Global health	−0.41	0.12	(−0.6, −0.1)	0.15
Upset of hair loss				
Family history of cancer	−6.08	2.56	(−11.15, −1.0)	0.15
Chronic disease	11.70	4.61	(2.57, 20.8)	0.18
Work stats	9.64	4.82	(0.11, 19.1)	0.21
Hormonal therapy	23.79	6.22	(11.48, 36.1)	0.08
**Satisfaction-**Nurse -technical skills	−0.19	0.08	(−0.35, −0.0)	0.12

**1. Method=stepwise:** Place of residence age, marital status, education, job, number of children, family size, income, stage of disease, chemotherapy, radiotherapy, hormonal therapy, biological therapy, type of surgical intervention, chronic diseases, pain medications, family history of cancer, family support, husband support, other sources of support, physical functioning, role functioning, emotional functioning, cognitive functioning, social functioning, fatigue, nausea and vomiting, pain, dyspnea, insomnia, appetite loss, constipation, diarrhea, financial difficulties, global health, doctors’ technical skills, interpersonal skills, information provision, availability, nurses’ technical skills, nurses’ interpersonal skills, information provision, availability, information exchange, kin help provision, waiting time, access to services, hospital comfort and cleanliness, and overall satisfaction.

**2. Method=stepwise:** Body image, sexual functioning, sexual enjoyment, future perspective, systemic therapy side effect, breast symptom, arm symptoms, upset of hair loss.

#### 3.6.1 QoL body image domain

Age exhibited a positive association with body image (*B* = 22.61, 95% CI: 14.6 to 30.5), suggesting a potential tendency for older individuals to report a more positive perception of their body image, although the model explained a modest proportion of the variance (*R*^2^ = 0.21). Notably, support from the husband demonstrated a strong and significant positive effect on body image (*B* = 11.80, 95% CI: 3.5 to 20.0, *R*^2^ = 0.47), underscoring the crucial role of spousal support in this domain. Conversely, surgical intervention was associated with a negative impact on body image (*B* = −15.03, 95% CI: −25.1 to −4.1, *R*^2^ = 0.30), while receiving biological treatment correlated with better body image (*B* = 11.75, 95% CI: 4.1 to 19.3, *R*^2^ = 0.40). Furthermore, lower role functioning (QoL-Role Functioning) was linked to poorer body image (*B* = −0.25, 95% CI: −0.4 to 0.0, *R*^2^ = 0.44), and greater satisfaction with doctors’ interpersonal skills was associated with improved body image (*B* = 0.34, 95% CI: 0.2 to 0.4, *R*^2^ = 0.37) (see Table 6).

#### 3.6.2 QoL sexual functioning domain

Age was negatively associated with sexual functioning (*B* = −14.44, 95% CI: −20.4 to −8.4, *R*^2^ = 0.34), while being married showed a strong positive association (*B* = 33.89, 95% CI: 25.2 to 42.5, *R*^2^ = 0.23). Receiving biological treatment negatively affects sexual functioning (*B* = −9.51, 95% CI: −15.5 to −3.4, *R*^2^ = 0.43). Conversely, better global health QoL was associated with improved sexual functioning (*B* = 0.199, 95% CI: 0.003 to 0.39, *R*^2^ = 0.47). Fatigue emerged as a negative predictor (*B* = −8.29, 95% CI: −15.0 to −1.5, *R*^2^ = 0.39), and satisfaction with doctors’ interpersonal skills again demonstrated a beneficial effect (*B* = 0.19, 95% CI: 0.01 to 0.3, *R*^2^ = 0.46) (see Table 6).

#### 3.6.3 QoL-sexual enjoyment domain

Older age was associated with reduced sexual enjoyment (*B* = −13.66, 95% CI: −21.1 to −6.1, *R*^2^ = 0.25), while being married was linked to increased enjoyment (*B* = 26.50, 95% CI: 15.5 to 37.4, *R*^2^ = 0.17). Interestingly, and perhaps counterintuitively, the husband’s support was associated with reduced enjoyment (*B* = −13.92, 95% CI: −22.1 to −5.7, *R*^2^ = 0.31), a finding that warrants further exploration. Interestingly, our findings revealed a negative association between husbands’ support and women’s sexual enjoyment. This may be explained by the complex emotional and relational changes that occur following a cancer diagnosis. In some cases, women may perceive their husband’s support as a reminder of their illness or as a shift in the dynamics of their relationship, which can adversely affect intimacy. In other instances, support may be experienced as a sense of obligation rather than genuine affection, leading to feelings of emotional distance or disconnection. These complexities highlight the importance of gaining a deeper understanding of how women perceive and experience spousal support, particularly in relation to body image and intimacy during cancer treatment.

Positive cognitive functioning and global health were associated with increased enjoyment (*B* = 0.13 and 0.29, respectively; *R*^2^ = 0.45 and 0.34). Moreover, gastrointestinal symptoms (diarrhea) had a small positive effect (*B* = 0.12, 95% CI: 0.01 to 0.2, *R*^2^ = 0.43). Satisfaction with doctor availability had a negative effect (*B* = −0.30, 95% CI: −0.46 to −0.1, *R*^2^ = 0.362), while satisfaction with information exchange showed a positive relationship (*B* = 0.26, 95% CI: 0.1 to 0.4, *R*^2^ = 0.38).

#### 3.6.4 QoL future perspective domain

Husband support contributed positively to women’s future perspective (*B* = 7.76, 95% CI: 0.3 to 15.2, *R*^2^ = 0.54). Pain and receiving chemotherapy (*B* = 23.46, 95% CI: 2.0 to 44.8, *R*^2^ = 0.53) were identified as significant positive predictors of future perspective (*B* = 0.32, 95% CI: 0.1 to 0.5, *R*^2^ = 0.44), whereas biological treatment (*B* = −11.43, 95% CI: −18.4 to −4.4, *R*^2^ = 0.47) and having a chronic disease (*B* = −10.14, 95% CI: −17.6 to −2.6, *R*^2^ = 0.49) were negatively associated with patients’ future perspectives. Conversely, patients had a positive future perspective. Among QoL, better scores in nausea/vomiting (*B* = 0.47, *R*^2^ = 0.32), insomnia (*B* = 0.21, *R*^2^ = 0.41), and fewer financial difficulties (*B* = −0.12, *R*^2^ = 0.51) were associated with more optimistic future perspectives (see Table 6).

#### 3.6.5 QoL systemic therapy side effects

Age was positively associated with systemic therapy side effects (*B* = 12.57, 95% CI: 6.8 to 18.3, *R*^2^ = 0.22), while global health QoL was negatively associated (*B* = −0.41, 95% CI: −0.5 to −0.2, *R*^2^ = 0.11). Satisfaction with hospital comfort/cleanliness negatively predicted side effects (*B* = −0.24, 95% CI: −0.3 to −0.1, *R*^2^ = 0.25), and access to services had a small positive effect (*B* = 0.25, 95% CI: 0.01 to 0.4, *R*^2^ = 0.30) (see Table 6).

#### 3.6.6 QoL breast and arm symptom

Breast symptoms were negatively influenced by a lack of family support (*B* = −18.39, 95% CI: −29.4 to −7.3, *R*^2^ = 0.16), while surgical intervention was a positive predictor (*B* = 9.50, 95% CI: 1.7 to 17.2, *R*^2^ = 0.20). QoL related to pain had a weak positive effect (*B* = 0.30, *R*^2^ = 0.11), and comfort/cleanliness satisfaction again showed a negative association (*B* = −0.22, *R*^2^ = 0.05). For arm symptoms, surgical intervention was a major positive predictor (*B* = 22.26, *R*^2^ = 0.08), followed by biological treatment (*B* = 10.69, *R*^2^ = 0.200). Lower education level also contributed to increased symptoms (*B* = −6.32, *R*^2^ = 0.22), and global health again showed a negative relationship (*B* = −0.41, *R*^2^ = 0.15) (see Table 6).

#### 3.6.7 QoL upset of hair loss

Upset due to hair loss was significantly associated with personal and clinical characteristics. A family history of cancer (*B* = −6.08, 95% CI: −11.15 to −1.0, *R*^2^ = 0.15) and having a chronic disease (*B* = 11.70, *R*^2^ = 0.18) were both predictors of upset of hair loss. Employment status also played a role (*B* = 9.64, *R*^2^ = 0.21), and hormonal therapy was strongly associated with more upset (*B* = 23.79, *R*^2^ = 0.08). Additionally, dissatisfaction with nurses’ technical skills was negatively associated with this upset (*B* = −0.197, *R*^2^ = 0.12) (Table 6).

## 4 Discussion

This study aimed to assess the quality of life (QoL) and patient satisfaction among newly diagnosed Palestinian women with breast cancer living in the southern region of the West Bank. At diagnosis, physical and cognitive functioning were relatively high, while social functioning was the lowest. Breast cancer symptoms like fatigue, insomnia, and dyspnea were moderate, with diarrhea and nausea/vomiting being the least reported. Global health status indicated a moderate QoL at the start of treatment. One year after treatment, body image concerns were moderate, while sexual functioning, sexual enjoyment, and future perspective were lower, especially regarding uncertainty about the future. Symptoms related to therapy and physical aspects were low. Doctors’ technical skills received high ratings, but their interpersonal skills and information provision were moderate. Nurses’ technical skills were also well-rated, but their availability and interpersonal skills were less satisfactory. Waiting time and access to care received the lowest satisfaction scores, highlighting areas for improvement. Moreover, the current findings offer nuanced insights into the multidimensional aspects of quality of life (QoL) among women undergoing cancer treatment, particularly in domains related to body image, sexual health, psychosocial support, treatment side effects, and personal well-being. Our results reflect the women’s experiences of a fragmented healthcare system, societal stigma, and limited psychosocial support in the Palestinian context. The Palestinian healthcare system faces ongoing and complex challenges (Rosenthal, 2021). Political instability, treatment availability, limited resources, insufficient health research, and referral restrictions are all barriers to improving cancer care in Palestine (AlWaheidi, 2019). Furthermore, the lack of standardized protocols for breast cancer care and the absence of multidisciplinary care teams further negatively impact patients’ quality of life and satisfaction (Abo Al-Shiekh et al., 2019).

### 4.1 Quality of life at diagnosis using EORTC QLQ-C30

The QoL among newly diagnosed breast cancer patients using the EORTC QLQ-C30 revealed notable variations across functional and symptom domains. Physical and cognitive functioning had the highest mean scores, suggesting a relatively preserved ability to perform daily tasks and maintain mental clarity at the time of diagnosis. In contrast, social functioning recorded the lowest score, reflecting significant challenges in social roles and relationships. The sociocultural stigma surrounding cancer, particularly breast cancer, often leads to isolation and fear of judgment. Also, being a breast cancer patient with its associated fatigue and emotional toll, naturally hinders social engagement, making it harder for patients to maintain pre-diagnosis relationships and roles. In addition, our findings showed that global health status was moderately rated, which was lower than the results reported in Brazil (dos Santos et al., 2023), India (Shinde et al., 2020), and Iraq (Rashid et al., 2022; Saleh and Narjes, 2021). This reflects a relatively lower perception of overall health and QoL among breast cancer patients in Palestine, possibly due to cultural, economic, or healthcare system factors.

This could be related to the sociocultural stigma, which postpones diagnosis and leads to isolation, and the fragmented and inadequately supported healthcare system, which may hinder access to timely and specialized treatments such as radiation or essential medications, requiring complex permit processes for seeking care elsewhere.

Also, our study participants reported lower functional abilities, particularly in emotional functioning, which aligns with the Sudanese study (mean 93.97) (Himedan and Hassan, 2017) but is notably lower than findings from an Indonesian study, where the mean scores for functional and cognitive functioning were 87.6 and 90.6, respectively(Yuliawati et al., 2024). When compared to other local studies, our participants showed higher mean scores in physical functioning (Gaza Stri*p* = 60, West Bank = 48.5), but lower mean scores in emotional functioning (Gaza Stri*p* = 64, West Bank = 77.8) (Maraqa and Ahmead, 2021; ElMokhallalati et al., 2022). This may reflect the deterioration in the Palestinian social and psychological support and healthcare services that have been going on over the past 2 years in Palestine due to the war atmosphere in the region. The symptom scales further illustrate the patients’ burden, with fatigue, insomnia, dyspnea, and pain emerging as the most significant complaints. These symptoms are consistent with findings from Bosnia and Herzegovina (Gavric and Vukovic-Kostic, 2015), Brazil (Costa et al., 2017; Marcelo Silva and Lúcia, 2021), and other Arab countries such as Tunisia, Egypt, and Saudi Arabia (Imran et al., 2019; Charfi et al., 2018; Shimozuma, 2022). These findings support prior conclusions by Ganz (2006) and Quattropani et al. (2017) that breast cancer and its treatments have complex and far-reaching consequences on patients’ lives. Addressing these challenges requires comprehensive care that integrates medical with psychosocial treatment and rehabilitative interventions to improve overall QoL (Ganz, 2006; Quattropani et al., 2017). Similarly, local studies in Gaza and the West Bank also identified fatigue, loss of appetite, and pain as the most troubling symptoms (Maraqa and Ahmead, 2021; ElMokhallalati et al., 2022). These symptoms significantly compromise QoL by limiting patients’ ability to engage socially and emotionally, highlighting the importance of early symptom control and supportive care. In summary, we can justify these findings on QoL at diagnosis, i.e., poor emotional and social functioning scores, by a feeling of uncertainty and loneliness, which can be related to the limited psychosocial support, fear of stigma, and cultural pressure. This emphasizes the critical need for emotional support services and community-based awareness campaigns to enhance early QoL outcomes in this population.

### 4.2 Patient satisfaction with breast cancer care

Patient satisfaction is a key indicator of healthcare quality and plays a critical role in shaping treatment adherence and overall health outcomes (Manjali et al., 2020). Regarding satisfaction of care, our findings align with global research, which demonstrates considerable variation in satisfaction levels across different countries. In our study, overall patient satisfaction with healthcare services was 45.60%. Among the various aspects of care, the highest satisfaction levels were reported for the technical skills of doctors (68.13%) and nurses (63.27%). In contrast, the lowest satisfaction levels were observed in access to care and waiting times, with scores of 35.42 and 33.68%, respectively. Our findings are consistent with an Egyptian study, which reported that 56.4% of breast cancer patients were satisfied with healthcare services. Similar to our results, doctors’ and nurses’ interpersonal and technical skills received the highest satisfaction scores, while nurses’ availability, information exchange, comfort, and cleanliness received the lowest satisfaction scores (Abdelsalam and Bayomi, 2022). Similarly, in Ethiopia, the overall patient satisfaction score was 44.8%. Patients reported higher satisfaction with doctors’ availability, interpersonal skills, and technical competence (50.2–52.6%) compared to nurses’ responsiveness and information provision (46.2%). The lowest-rated aspects of healthcare in Ethiopia included waiting times for medical tests and treatments, as well as the kindness and helpfulness of technical, reception, and laboratory staff (Abate et al., 2020). These patterns reflect broader international findings. For instance, in France, patient satisfaction scores ranged from 61 for doctors’ availability to 78 for nurses’ interpersonal skills, with higher satisfaction for doctors’ information provision compared to overall cancer patient data (Brédart et al., 2013). In Slovakia, patient satisfaction was primarily driven by nursing care, with the highest scores for nurses’ interpersonal skills, technical competence, and information provision. However, doctors’ availability, waiting times, access, and comfort were rated lower (Obročníková and Majerníková, 2017). Similarly, in another study, overall patient satisfaction was moderate, with an average score of 51.

Although doctors’ and nurses’ technical skills received ratings above 50, aspects such as waiting times (42.76%) and hospital access (28.95%) were reported as the least satisfactory (Obtel et al., 2017). In Poland, an overall satisfaction rate of 68.4% was found, with higher ratings for doctors’ technical skills (66.7%) and nurses’ technical skills (70.8%), while hospital access, waiting time, and information exchange each received lower ratings of 50% (Skręt-Magierło et al., 2016). Higher satisfaction scores were reported in Italy (88.52%) (Djambazov and Giammanco, 2019) and Poland (81.73%) (Rudzińska et al., 2025) reflecting strong healthcare performance in these regions, particularly in physician and nursing care. In contrast, moderate satisfaction levels were observed in Iran (59.43%) (Pishkuhi et al., 2014), Switzerland (67.3%) (Marino et al., 2021), and Mexico (72.30%) (Balderas-Peña et al., 2019), suggesting room for improvement in certain aspects of service delivery.

Our findings highlight the need for healthcare improvements, particularly in accessibility, waiting times, and communication, to enhance patient satisfaction in breast cancer. Long waiting times and limited access to care are likely due to resource shortages and referral delays. Poor communication may be caused by stressed employees and a lack of training. Patients’ trust and adherence may suffer due to these variables, underscoring the need for more responsive and patient-centered treatment in this context. Also, patients with breast cancer in Palestine frequently complain about the quality of healthcare services because of a fragmented and disorganized system that is made worse by political limitations that seriously delay or prevent them from receiving necessary specialized treatments like radiotherapy or necessary drugs.

### 4.3 Quality of life using EORTC QLQ-C23 after one year of treatment

In comparison to other countries, the mean body image score in our study (mean = 43.17) was lower than in Iraq (Rashid et al., 2022; Saleh and Narjes, 2021), Brazil (dos Santos et al., 2023; Campos et al., 2024), Saudi Arabia (Imran et al., 2019), and Pakistan (Malik et al., 2020), but higher than in Iran (Tahmasebi-Ghorrabi et al., 2024) and Poland (Słowik et al., 2017). Similarly, Omari et al. indicated that a considerable percentage of Palestinian women with breast cancer had body image dissatisfaction, 43.1% were dissatisfied, and 58.8% did not see their body image as attractive, despite 74% valuing their appearance. Dissatisfaction was much greater among those getting major surgeries, intense therapies, and advanced-stage treatments. These findings emphasize the necessity for healthcare providers to prioritize patient satisfaction by delivering clear education on treatment alternatives and probable results, fostering open dialogues regarding body image issues, and ensuring access to psychosocial support, reconstructive options, and lifestyle interventions intended to improve self-perception and overall quality of life (Omari et al., 2024).

Sexual functioning (mean = 34.49) and sexual enjoyment (mean = 34.72) in our study were comparable to Brazil (dos Santos et al., 2023), lower than Iraq, Saudi Arabia, and Egypt (Rashid et al., 2022; Saleh and Narjes, 2021; Imran et al., 2019; Shimozuma, 2022), but higher than Iran (Tahmasebi-Ghorrabi et al., 2024) and Poland (Konieczny and Fal, 2023). The future perspective domain (mean = 23.38) recorded the lowest score among all compared studies, reflecting significant uncertainty about life after treatment. Regarding symptom domains, systemic therapy side effects (mean = 26.22) were lower than those in Iraq, Saudi Arabia, and Egypt (Saleh and Narjes, 2021; Imran et al., 2019; Shimozuma, 2022) but higher than in Brazil (da Silva et al., 2022) and Pakistan (Malik et al., 2020). Meanwhile, arm symptoms (mean = 37.19) and breast symptoms (mean = 33.33) were higher than reported in Iraq (Saleh and Narjes, 2021) and Brazil (Barbosa et al., 2017), but similar to Saudi Arabia (Imran et al., 2019).

Many women faced ongoing sexual challenges influenced by both treatment effects and social taboos, alongside deep concerns about their future. Differences in symptom scores across countries suggest variability in care quality. These results underscore the need for enhanced post-treatment support and open communication.

Upset by hair had a mean score of just 14.81, which was significantly lower than in Iraq (Rashid et al., 2022), Brazil (dos Santos et al., 2023), Poland (Konieczny and Fal, 2023), and Egypt (Shimozuma, 2022). The low score for distress about hair loss likely reflects that most women had completed chemotherapy by the time of data collection and had begun to regrow their hair. Over the years, many may have emotionally adapted to this change, turning their focus to concerns regarding body image, relationships, and long-term health.

### 4.4 Determinants of quality of life post-treatment and association with patients’ satisfaction

#### 4.4.1 Body image

Represents a key dimension of quality of life in women diagnosed with breast cancer. Findings from this study indicate that older age and strong spousal support act as protective factors, helping women adjust more effectively to appearance-related changes. However, an inverse relationship was found between age and both sexual functioning and enjoyment, indicating that older patients are particularly vulnerable in this domain. Consistent with our findings, several other studies have shown that women in the younger age group tend to have lower QoL, particularly about body image and concerns about future health, compared to older women. For example, a study conducted in Singapore found that younger women reported more concerns regarding nausea, body image, and future health than their older counterparts (Tan et al., 2014; Campos et al., 2024). These findings are further supported by previous research indicating that younger women often experience poorer QoL following treatment, especially in domains related to cognitive functioning, sexual functioning, sexual enjoyment, and body image (Kostic et al., 2020; dos Santos et al., 2023; Omari et al., 2024). A possible explanation is that older patients tend to be more emotionally mature, which may enhance their ability to cope with breast cancer. In contrast, younger patients may require additional emotional support, particularly in dealing with changes in body image and interpersonal relationships.

In addition, surgical treatment was significantly associated with lower body image scores and increased breast and arm symptoms, which negatively impacted body image, sexual relations, and social activities. These results suggest that women who underwent surgery experienced greater dissatisfaction with their physical appearance post-treatment. These findings are consistent with prior research indicating that younger patients often experience a decline in QoL following surgery, with mastectomy having a particularly negative effect on body image (Kostic et al., 2020; Słowik et al., 2017; Papadopoulos et al., 2011). According to Barbosa et al. (2017) and Konieczny and Fal (2023), women’s QoL is influenced by the type of surgical treatment they receive for breast cancer. Therefore, whenever feasible, the chosen method should aim to preserve the breast or allow for its reconstruction after surgery (Konieczny and Fal, 2023; Barbosa et al., 2017). The literature shows that satisfaction with breast surgery has been strongly associated with better psychological outcomes and long-term QoL (Shiraishi et al., 2022; Ghilli et al., 2023). Krzos et al. (2019) and Stolpner et al. (2021) found that higher satisfaction following breast-conserving surgery was linked to improved emotional and social functioning, despite minor declines in physical well-being. Lower satisfaction, on the other hand, was associated with poorer baseline psychosocial health (Krzos et al., 2019; Stolpner et al., 2021). Similarly, Baumbach et al. (2023) reported that satisfaction with physician and nurse care was positively correlated with QoL and self-rated health (Baumbach et al., 2023). A 5-year scoping review by Saiga et al. (2023) emphasized that both patient satisfaction and QoL are influenced not only by surgical outcomes, but also by cultural, social, and methodological factors (Saiga et al., 2023). Therefore, we can justify the high satisfaction of our participants with their doctors’ interpersonal qualities, such as showing empathy, listening attentively, and communicating respectfully, as a very important factor for a positive body image among breast cancer women. These associations suggest that supportive, patient-centred interactions can play a vital role in how women see themselves after undergoing breast cancer treatment. Feeling heard and understood by healthcare providers may help ease body-related insecurities and promote a sense of self-acceptance during recovery. Several studies indicate that patients are dissatisfied with the information provided by physicians due to inadequate communication (Ignatowicz et al., 2025; Longo et al., 2022).

#### 4.4.2 Sexual functioning and sexual enjoyment

Marital status emerged as an important factor influencing the QoL among breast cancer patients. Women who were married reported better sexual functioning and greater sexual enjoyment; being married appeared to offer some protective effect in both areas. Previous studies by Cobo-Cuenca et al. (2019) and Acil and Cavdar (2014) have highlighted that women in marital relationships reported higher levels of life satisfaction compared to their unmarried counterparts (Acil and Cavdar, 2014; Cobo-Cuenca et al., 2019). A potential explanation of these findings supports other studies that showed being in a supportive marital relationship can positively affect coping, intimacy, and overall well-being during cancer treatment (Ştefǎnuţ et al., 2021; Valente et al., 2021).

Regarding partner or family support, our findings underscore the significant role of partner support in enhancing the QoL among women with breast cancer. Being in a supportive relationship not only contributes to emotional well-being but also provides practical and psychological resources that can help patients better cope with the challenges of diagnosis and treatment. Family support, particularly from the husband/partner, plays a crucial role in improving resilience and treatment outcomes (Konieczny et al., 2020). According to Su et al. (2017) and da Silva et al. (2022), married women with breast cancer who receive support from their partners and families during treatment are less likely to develop major depressive disorder. This emotional support contributes positively to their psychological well-being and, in turn, enhances their overall QoL (da Silva et al., 2022; Su et al., 2017). Participants who received strong spousal support tended to have a more hopeful outlook regarding their future. However, in a somewhat unexpected finding, spousal support was negatively associated with sexual enjoyment. This contradiction may reflect unmet expectations or shifting relational dynamics within couples, particularly in the context of illness. These results highlight the importance of integrating family- or couple-based psychosocial interventions into care plans, particularly for younger patients who may be more vulnerable to body image disturbances.

Our data indicated that sexual enjoyment was negatively associated with patient satisfaction in doctor availability, implying that limited access to doctors may lead to discomfort or feelings of neglect, so impacting patients’ private lives. While, satisfaction with the exchange of information between patients and healthcare practitioners showed a positive association with sexual enjoyment. This underscores the importance of clear, sympathetic communication in influencing a woman’s well-being and self-confidence following breast cancer treatment. Several studies have highlighted the central role of communication in shaping patient satisfaction. For instance, a recent large-scale survey in China found that doctor–patient communication was one of the strongest predictors of outpatient satisfaction. Many patients expressed lower satisfaction when their expectations about interpersonal interactions were unmet. These findings emphasize the need for healthcare providers to enhance their communication skills as a key strategy to improve patient experiences and trust (Xintong et al., 2024).

#### 4.4.3 Systemic therapy side effect: breast, arm, hair loss symptoms

Among the most commonly reported symptoms in women with breast cancer were fatigue, insomnia, nausea and vomiting, diarrhea, and pain. These symptoms were not only physically distressing but also had a significant impact on patients’ emotional well-being, contributing to increased irritability and stress. Furthermore, these symptoms were closely linked to impairments in sexual functioning, reduced sexual enjoyment, negative future outlook, and an increase in breast-related symptoms. Several studies have consistently highlighted the crucial role these symptoms play in diminishing the overall QoL of breast cancer patients. For instance, research by Nageeti et al. (2019) and Shinde et al. (2020) identified fatigue, insomnia, nausea and vomiting, and pain (breast and arm) as some of the most influential factors negatively affecting QoL (Shinde et al., 2020; Nageeti et al., 2019). Similarly, findings by Konieczny et al. (2020) and Rashid et al. (2022) emphasized that women diagnosed with breast cancer who experienced these symptoms reported significantly lower levels of QoL. These symptoms made the patients feel worse physically and also increased their mental stress, making treatment and recovery more difficult (Rashid et al., 2022; Konieczny et al., 2020).

Regarding chronic diseases, our results showed that comorbidities were associated with a more negative outlook on the future among breast cancer patients. Older women reported a greater burden of systemic side effects, likely due to comorbidities or increased sensitivity to treatment.

Moreover, chronic diseases also contributed to greater emotional distress. This suggests that the presence of other health problems adds to the overall challenges of cancer treatment. These findings are consistent with previous studies; for example, in India, comorbidities such as diabetes, hypertension, and other chronic conditions significantly reduced QoL and posed major challenges in cancer care by increasing the overall burden and negatively affecting treatment response (Sebastian JJ and Mathews, 2023). Similar results were reported in studies conducted in the USA and Indonesia, where comorbidities were correlated with lower QoL among breast cancer patients (Yuliawati et al., 2024; Fu et al., 2015). Our results show how personal, relational, and clinical aspects all work together to affect the lives of Palestinian women after they have had breast cancer treatment. Chronic disease, limited access to rehabilitation treatments, cultural silence around sexual health, and not enough psychological support all make post-treatment quality of life worse. Tailored interventions must account for these realities to provide more holistic, culturally sensitive care. Additionally, findings show that the health system environment plays a key role in breast cancer patients’ experiences. Satisfaction with hospital cleanliness and comfort was linked to fewer reported side effects. This highlights how both the physical setting and service access shape patient satisfaction and perceptions of treatment. Prior research supports that clean, quiet, and well-designed environments improve emotional well-being and patients satisfaction (MacAllister et al., 2016).

Dissatisfaction with the nursing care, cleanliness, and comfort of the healthcare environment can profoundly exacerbate the physical and psychological burden of side effects experienced by breast cancer patients undergoing systematic therapy. When patients feel their nurses are unresponsive, lack empathy, or are inadequately skilled, their pain, whether generalized, neuropathic, or related to arm issues, may go unaddressed or undertreated, leading to increased suffering and a sense of abandonment. Similarly, a perceived lack of cleanliness or an uncomfortable environment (e.g., noisy wards, uncomfortable beds, poor temperature control) can amplify distress, making it harder for patients to cope with the emotional toll of hair loss and the physical discomfort of arm pain or generalized pain (Johnsson et al., 2023; Ikeda et al., 2020; Busari and Henry, 2025). This cumulative dissatisfaction creates a cycle where unmet basic needs and significantly reducing their overall quality of life during a vulnerable period.

In the Palestinian context, these findings are of particular importance. Many women face not only the physical and emotional challenges of breast cancer, but also additional burdens such as socio-political instability, limited access to specialized care, and deeply rooted cultural expectations around body image and sexuality. These characteristics can significantly influence women’s perceptions of their treatment experiences, self-identity, and overall quality of life. To effectively support survivors, care models must go beyond clinical outcomes to address emotional needs and provide culturally sensitive, patient-centred support throughout the cancer journey.

### 4.5 Implications

The findings of this study carry significant clinical and policy implications, emphasizing the need for a more holistic, patient-centred approach to breast cancer care. Clinically, the consistent impact of psychosocial factors such as spousal support, satisfaction with doctor–patient communication, and perceived hospital comfort highlights the importance of embedding psychological support, couple or family counseling, and empathetic communication training within routine oncology care. Tailored interventions should be prioritized to address body image concerns, sexual health challenges, and emotional well-being, particularly for older patients, those receiving invasive treatments, and individuals with coexisting chronic conditions.

From a policy standpoint, the results underscore the urgency of strengthening healthcare systems by improving service accessibility, maintaining high standards of hospital cleanliness and comfort, and investing in the training of healthcare professionals on interpersonal and psychosocial care skills. Furthermore, policymakers should advocate for the implementation of comprehensive survivorship care plans that extend beyond physical symptom management to encompass mental health support, social connectedness, and quality of life enhancement, particularly for vulnerable subgroups. Such integrated efforts are essential to improving the overall care experience and long-term outcomes for women diagnosed with breast cancer.

### 4.6 Limitations

This study has several limitations. First, although the cross-sectional design allowed for identifying correlations, it did not permit conclusions about causality. Second, the sample was limited to newly diagnosed breast cancer patients receiving treatment in governmental hospitals in the southern West Bank, which may affect the generalizability of the findings to patients in private healthcare settings or other geographic regions. Lastly, the impact of treatment modalities was not assessed, highlighting the need for future research to examine how different treatments influence women’s perceptions and satisfaction levels.

## 5 Conclusion

This study assessed the QoL and satisfaction with care among newly diagnosed Palestinian women with breast cancer over one year. At the time of diagnosis, the findings revealed a notably low level of QoL, particularly in the emotional and social functioning domains. Additionally, a high burden of symptoms, such as fatigue and pain, was observed, which were negatively associated with QoL. After one year of follow-up, significant variations in QoL were noted based on factors such as age, the presence of chronic illness, the type of surgical intervention, and the level of support received from partners and family members. The study also found a low level of satisfaction with the healthcare services provided in governmental hospitals, particularly regarding access, waiting times, information provision, and communication between patients and healthcare providers. Improving patient-centered care, particularly in sexual health, body image, and support services, is essential for enhancing patient experience and treatment outcomes in Palestine’s resource-limited and frequently unstable healthcare context. Healthcare services in Palestine should emphasize efficient symptom management, open communication, and enhanced psychosocial support networks for cancer patients, acknowledging the unique stressors and limited infrastructure characteristic of a conflict-affected area.

## Data Availability

The original contributions presented in the study are included in the article/Supplementary material, further inquiries can be directed to the corresponding author/s.
