# Supplementary material for: Satisfaction and quality of life of Palestinian women with newly diagnosed breast cancer: a one-year follow-up study
Source: Front Psychol. 2025 Sep 22;16:1614851. doi: 10.3389/fpsyg.2025.1614851 (PMC12499494; doi:10.3389/fpsyg.2025.1614851)
Supplement: Supplementary file 1 [file Data_Sheet_1.pdf]

Table S1: EORTC QLQ-C30 Functional scales and socio-demographic, clinical and social support variables (N=144)

Variables	Physical function	Role function	Emotional function	Cognitive function	Social function
	Mean rank	Mean rank	Mean rank	Mean rank	Mean rank
**Residence**					
Refugee camp	62.14	63.95	42.45	63.95	68.73
Village	72.51	74.53	77.99	71.98	70.36
City	75.13	70.44	68.70	75.78	77.95
**P-value**	0.65	0.64	**0.02**	0.68	0.57
**Age in years**					
≤ 40	84.65	83.05	64.49	78.59	86.03
41–54	59.29	61.04	81.20	65.88	57.80
**P-value**	**0.001**	**0.001**	**0.01**	0.06	**0.001**
**Marital status**					
Unmarried	69.55	80.73	67.08	64.38	20.00
Married	72.98	71.17	73.38	73.81	80.97
**P-value**	0.73	0.30	0.52	0.34	**0.001**
**Education**					
Primary and less	53.79	63.89	89.47	58.29	47.50
Secondary	73.48	71.72	68.75	73.16	72.73
Colleague/University	76.96	75.61	70.93	75.96	79.39
**P-value**	0.09	0.50	0.15	0.25	**0.01**
**Working status**					
Employee	86.44	72.15	75.73	79.95	85.50
Housewife	66.95	72.64	71.21	69.53	67.33
**P-value**	**0.01**	0.94	0.55	0.16	**0.01**
**Number of children**					
≤5 persons	76.32	73.30	72.47	77.06	73.22
>5 persons	64.37	70.79	72.57	62.79	70.97
**P-value**	0.10	0.71	0.99	0.05	0.75
**Family size**					
≤5 persons	75.95	74.42	75.19	78.95	67.50
>5 persons	70.61	71.45	71.03	68.96	75.24
**P-value**	0.46	0.66	0.56	0.16	0.27
**Income (US $)**					
< 570	61.73	64.23	69.70	65.53	70.00
570-1140	76.64	77.03	73.97	74.29	72.13
> 1140	82.23	77.45	74.30	80.47	77.27
**P-value**	0.05	0.15	0.82	0.24	0.72
**Stage at diagnosis**					
Stage 1	87.32	65.09	69.27	70.14	81.86
Stage 2	71.20	70.16	64.09	70.86	69.34
Stage 3	73.17	75.05	76.02	72.70	75.25
Stage 4	59.40	77.70	101.90	83.30	62.60
**P-value**	0.48	0.78	**0.04**	0.84	0.61
**Chemotherapy**					
Yes	70.92	72.16	72.83	71.60	72.09
No	127.88	84.38	61.13	103.88	86.88
**P-value**	**0.007**	0.53	0.57	0.12	0.47
**Radiotherapy**					
Yes	75.77	72.36	71.83	76.23	74.00
No	63.69	72.88	74.31	62.45	68.47
**P-value**	0.12	0.94	0.74	0.07	0.47
**Hormonal therapy**					
Yes	72.41	73.21	74.43	74.85	74.22
No	73.05	68.33	61.21	58.71	62.40
**P-value**	0.94	0.59	0.17	0.09	0.22
**Biological treatment**					
Yes	76.78	70.43	63.67	69.14	81.22
No	67.97	74.69	81.84	76.05	63.28
**P-value**	0.20	0.51	**0.008**	0.31	**0.009**
**Surgical intervention**					
Complete Mastectomy	66.28	69.04	73.03	65.22	64.97
Partial Mastectomy	76.69	74.83	72.14	77.41	77.58

**P-value**	0.14	0.38	0.89	0.08	0.07
**Chronic disease**					
Yes	53.30	61.26	77.74	63.54	52.56
No	81.80	77.95	69.96	76.84	82.16
**P-value**	**0.001**	0.01	0.28	0.06	**0.001**
**Pain Medication**					
Yes	67.11	69.78	68.72	70.65	64.71
No	80.26	76.42	77.94	75.16	83.73
**P-value**	0.06	0.31	0.18	0.51	**0.006**
**Relatives cancer**					
Breast Cancer	75.33	72.99	59.83	68.79	77.78
Other Cancers	64.09	69.27	80.47	68.41	73.73
No history of cancer	75.13	73.86	75.80	76.72	68.82
**P-value**	0.38	0.84	0.06	0.49	0.53
**Family support**					
Yes	72.42	72.63	72.28	72.79	74.11
No	73.00	71.68	73.85	70.73	62.53
**P-value**	0.95	0.91	0.87	0.83	0.24
**Husband support**					
Yes	80.91	72.00	68.41	80.93	89.88
No	68.80	72.72	74.30	68.79	64.86
**P-value**	0.10	0.91	0.43	0.10	**0.001**
**Other support**					
Yes	73.44	72.88	79.42	72.08	68.62
No	72.29	72.42	70.97	72.59	73.36
**P-value**	0.89	0.95	0.34	0.95	0.59

Significant p-value at $p < 0.05$ (2 tailed).

Table S2: EORTC QLQ-C30 Symptom scales and socio-demographic, clinical and social support variables (N=144)

Variables	Fatigue	Nausea/ Vomiting	Pain	Dyspnoea	Insomnia
	Mean rank	Mean rank	Mean rank	Mean rank	Mean rank
**Residence**					
Refugee camp	91.18	76.77	80.00	62.41	84.77
Village	69.93	68.01	69.84	76.43	67.75
City	73.10	80.80	76.15	66.85	79.30
**P-value**	0.27	0.16	0.57	0.29	0.17
**Age in years**					
≤ 40	68.74	77.39	66.79	62.04	73.78
41–54	76.59	67.18	78.71	83.87	71.11
**P-value**	0.25	0.09	0.07	**0.001**	0.69
**Marital status**					
Unmarried	75.25	82.28	73.50	77.80	74.88
Married	72.06	70.92	72.34	71.65	72.12
**P-value**	0.748	0.205	0.90	0.52	0.77
**Education**					
Primary and less	84.63	63.45	86.00	90.61	77.32
Secondary	73.40	70.67	76.30	71.24	74.15
Colleague/University	68.28	76.65	65.38	68.46	69.71
**P-value**	0.30	0.34	0.09	0.09	0.70
**Working status**					
Employee	62.65	74.52	61.51	68.82	66.02
Housewife	76.42	71.69	76.87	73.97	75.08
**P-value**	0.07	0.68	**0.04**	0.48	0.22
**Number of children**					
≤5 persons	68.67	71.70	71.46	67.69	73.27
>5 persons	80.65	74.21	74.72	82.75	70.86
**P-value**	0.10	0.70	0.65	**0.03**	0.73
**Family size**					
≤5 persons	70.85	75.86	71.34	68.70	74.74
>5 persons	73.40	70.66	73.13	74.59	71.27
**P-value**	0.72	0.42	0.80	0.39	0.62
**Income (US $)**					
< 570	77.89	73.23	84.13	80.38	83.40
570-1140	68.59	76.08	64.44	69.93	63.78
> 1140	71.06	64.61	68.70	64.52	71.13
**P-value**	0.48	0.36	**0.03**	0.17	**0.03**
**Stage at diagnosis**					
Stage 1	75.05	84.00	70.41	69.64	86.05
Stage 2	76.09	73.84	74.92	74.33	71.84
Stage 3	69.02	69.68	69.46	73.66	71.78
Stage 4	71.50	70.45	80.50	57.50	66.15
**P-value**	0.81	0.67	0.80	0.65	0.67
**Chemotherapy**					
Yes	72.30	72.72	71.77	72.75	72.50
No	79.50	64.75	98.13	63.88	72.63
**P-value**	0.73	0.67	0.20	0.66	0.99
**Radiotherapy**					
Yes	73.26	71.21	70.55	71.59	71.59
No	70.46	75.96	77.76	74.96	74.95
**P-value**	0.71	0.49	0.34	0.65	0.65
**Hormonal therapy**					
Yes	71.66	70.14	72.17	71.44	72.39
No	77.43	86.33	74.43	78.71	73.14
**P-value**	0.55	0.06	0.81	0.44	0.93
**Biological treatment**					
Yes	73.43	73.91	71.89	74.76	76.39
No	71.51	71.01	73.15	70.11	68.39
**P-value**	0.78	0.63	0.85	0.48	0.23
**Surgical intervention**					
Complete Mastectomy	78.34	75.57	78.22	72.41	75.16
Partial Mastectomy	68.56	70.43	68.65	72.56	70.70

**P-value**	0.16	0.41	0.16	0.98	0.51
Chronic disease					
Yes	82.18	74.57	78.13	85.87	71.98
No	67.81	71.49	69.77	66.02	72.75
**P-value**	0.05	0.64	0.24	**0.005**	0.91
Pain Medication					
Yes	80.10	72.89	81.46	78.39	77.68
No	61.55	71.93	59.59	64.01	65.03
**P-value**	**0.008**	0.87	**0.002**	**0.03**	0.06
**Relatives cancer**					
Breast Cancer	79.64	74.60	79.86	65.26	75.86
Other Cancers	69.80	75.06	72.77	78.54	62.69
No history of cancer	69.73	69.99	68.09	73.63	75.53
**P-value**	0.43	0.73	0.34	0.33	0.25
**Family support**					
Yes	73.15	72.04	72.63	71.06	74.50
No	68.48	75.33	71.73	81.40	60.13
**P-value**	0.63	0.71	0.92	0.28	0.13
**Husband support**					
Yes	75.90	76.97	69.95	63.97	68.89
No	71.01	70.54	73.62	76.26	74.09
**P-value**	0.51	0.33	0.61	0.09	0.47
**Other support**					
Yes	66.54	73.62	67.52	63.85	62.23
No	73.81	72.25	73.60	74.41	74.76
**P-value**	0.41	0.86	0.49	0.22	0.15

Significant p-value at $p < 0.05$ (2 tailed).

Table S3: EORTC QLQ-C30 Symptom scales and socio-demographic, clinical and social support variables (N=144)

Variables	Appetite Loss	Constipation	Diarrhoea	Financial Difficulties	Global health status
	Mean rank	Mean rank	Mean rank	Mean rank	Mean rank
**Residence**					
Refugee camp	73.59	74.23	65.73	71.14	40.32
Village	67.49	70.61	73.38	76.07	75.35
City	82.71	76.01	72.38	65.37	74.77
**P-value**	0.71	0.91	0.49	0.11	0.96
**Age in years**					
≤ 40	71.38	72.81	74.46	74.57	72.63
41–54	73.72	72.17	70.37	70.40	72.36
**P-value**	0.71	0.91	0.49	0.11	0.96
**Marital status**					
Unmarried	77.33	76.00	69.30	69.28	65.68
Married	71.72	71.94	73.02	73.02	73.60
**P-value**	0.53	0.64	0.66	0.69	0.42
**Education**					
Primary and less	68.45	69.29	84.00	80.05	53.97
Secondary	73.99	70.71	65.48	75.72	67.36
Colleague/University	72.36	74.96	75.31	67.57	82.20
**P-value**	0.85	0.73	0.09	0.35	**0.01**
**Working status**					
Employee	70.22	67.26	77.71	64.68	81.16
Housewife	73.41	74.59	70.43	75.61	69.05
**P-value**	0.64	0.27	0.26	0.13	0.11
**Number of children**					
≤5 persons	71.59	71.03	72.72	69.01	71.71
>5 persons	74.43	75.63	72.03	79.93	74.17
**P-value**	.674	.475	.914	.124	.738
**Family size**					
≤5 persons	75.60	68.79	73.97	70.59	74.90
>5 persons	70.80	74.53	71.69	73.55	71.18
**P-value**	0.46	0.36	0.71	0.66	0.60
**Income (US $)**					
< 570	73.77	77.04	78.92	83.24	63.54
570-1140	72.34	65.43	67.35	68.05	76.77
> 1140	70.73	78.38	71.72	63.39	79.06
**P-value**	0.93	0.13	0.22	**0.04**	0.14
**Stage at diagnosis**					
Stage 1	89.50	56.95	72.27	86.23	86.23
Stage 2	72.23	77.78	71.82	72.19	72.19
Stage 3	66.68	71.19	73.04	69.54	69.54
Stage 4	80.00	67.50	73.20	78.45	78.45
**P-value**	**0.03**	0.31	0.99	0.59	0.42
Chemotherapy					
Yes	71.57	72.60	73.24	73.22	72.13
No	105.13	69.00	46.50	47.25	85.38
**P-value**	0.08	0.84	0.13	0.19	0.52
Radiotherapy					
Yes	70.42	70.85	74.00	72.73	72.24
No	78.10	76.94	68.46	71.87	73.21
**P-value**	0.27	0.36	0.40	0.90	0.90
Hormonal therapy					
Yes	70.03	73.52	71.33	72.83	72.49
No	86.98	66.55	79.36	70.55	72.55
**P-value**	0.05	0.41	0.34	0.80	0.99
Biological treatment					
Yes	79.51	74.01	72.28	72.07	68.29
No	65.09	70.90	72.73	72.96	76.95
**P-value**	**0.02**	0.60	0.94	0.89	0.20
**Surgical intervention**					
Complete Mastectomy	74.47	73.33	76.53	76.84	62.24
Partial Mastectomy	71.17	71.94	69.78	69.57	79.42

**P-value**	0.608	0.821	0.266	0.282	0.014
**Chronic disease**					
Yes	78.34	75.67	73.50	78.97	68.70
No	69.67	70.96	72.02	69.37	74.34
**P-value**	0.19	0.46	0.81	0.17	0.44
**Pain Medication**					
Yes	78.19	76.16	73.54	73.93	68.19
No	64.31	67.23	71.01	70.44	78.70
**P-value**	**0.03**	0.14	0.67	0.60	0.13
**Relatives cancer**					
Breast Cancer	78.69	72.60	70.99	69.55	75.55
Other Cancers	70.94	70.36	77.01	83.20	77.26
No history of cancer	69.70	73.53	71.09	68.78	68.32
**P-value**	0.47	0.91	0.69	0.18	0.49
**Family support**					
Yes	72.22	72.19	74.09	71.48	70.84
No	74.25	74.40	62.63	78.80	82.80
**P-value**	0.82	0.79	0.18	0.44	0.22
**Husband support**					
Yes	67.50	71.50	66.10	70.65	79.35
No	74.70	72.94	75.32	73.32	69.49
**P-value**	0.29	0.82	0.15	0.71	0.18
**Other support**					
Yes	67.29	73.42	70.04	72.85	85.35
No	73.65	72.30	73.04	72.42	69.67
**P-value**	0.43	0.88	0.69	0.96	0.07

Significant p-value at $p < 0.05$ (2 tailed).

Table S4: EORTC QLQ-C23 functional scales and socio-demographic, clinical and social support variables (N=144)

Variables	Body image	Sexual functioning-	Sexual enjoyment	Future perceptive
	Mean rank	Mean rank	Mean rank	Mean rank
**Residence**				
Refugee camp	54.77	68.73	72.14	73.59
Village	75.24	70.36	69.85	67.49
City	71.29	77.95	78.14	82.71
**P-value**	0.29	0.57	0.52	0.09
**Age in years**				
≤ 40	54.09	86.03	86.03	86.03
41–54	92.51	57.80	57.80	57.80
**P-value**	**0.000**	**0.000**	**0.001**	0.71
**Marital status**				
Unmarried	87.70	20.00	26.98	77.33
Married	70.05	80.97	79.84	71.72
**P-value**	**0.040**	**0.000**	**0.000**	0.53
**Education**				
Primary and less	99.95	47.50	48.87	68.45
Secondary	71.96	72.73	72.97	73.99
Colleague/University	65.19	79.39	78.80	72.36
**P-value**	**0.005**	**0.011**	**0.013**	0.85
**Working status**				
Employee	74.06	85.50	81.85	81.85
Housewife	71.88	67.33	68.78	68.78
**P-value**	0.77	0.01	0.07	0.64
**Number of children**				
≤5 persons	69.89	73.22	74.48	71.59
>5 persons	78.07	70.97	68.27	74.43
**P-value**	0.27	0.75	0.37	0.67
**Family size**				
≤5 persons	81.88	67.50	66.35	75.60
>5 persons	67.35	75.24	75.87	70.80
**P-value**	**0.04**	0.27	0.16	0.46
**Income (US $)**				
< 570	69.07	70.00	66.60	73.77
570-1140	72.64	72.13	73.53	72.34
> 1140	77.81	77.27	80.17	70.73
**P-value**	0.64	0.72	0.29	0.93
**Stage at diagnosis**				
Stage 1	71.82	81.86	74.36	78.50
Stage 2	78.36	69.34	72.40	72.23
Stage 3	70.45	75.25	73.82	66.68
Stage 4	52.60	62.60	62.50	80.00
**P-value**	0.30	0.61	0.86	**0.03**
**Chemotherapy**				
Yes	72.95	72.09	72.51	71.57
No	56.88	86.88	72.25	105.13
**P-value**	0.44	0.47	0.99	0.08
**Radiotherapy**				
Yes	74.43	74.00	72.95	70.42
No	67.31	68.47	71.28	78.10
**P-value**	0.36	0.47	0.82	0.27
**Hormonal therapy**				
Yes	71.15	74.22	72.95	70.03
No	80.43	62.40	69.86	86.98
**P-value**	0.34	0.22	0.73	0.05
**Biological treatmen**t				
Yes	65.56	81.22	79.51	79.51
No	79.84	63.28	65.09	65.09
**P-value**	**0.03**	**0.009**	**0.02**	**0.02**
**Surgical intervention**				
Complete Mastectomy	60.28	64.97	64.57	74.47
Partial Mastectomy	80.74	77.58	77.85	71.17
**P-value**	**0.004**	0.07	**0.04**	0.60

**Chronic disease**				
Yes	93.36	52.56	60.98	78.34
No	62.39	82.16	78.08	69.67
**P-value**	**0.001**	**0.001**	**0.01**	0.19
**Pain Medication**				
Yes	77.71	64.71	66.22	78.19
No	65.00	83.73	81.55	64.31
**P-value**	0.07	**0.006**	**0.02**	**0.03**
**Relatives cancer**				
Breast Cancer	71.65	77.78	76.55	78.69
Other Cancers	70.16	73.73	72.24	70.94
No history of cancer	74.18	68.82	70.28	69.70
**P-value**	0.88	0.53	0.72	0.47
**Family support**				
Yes	72.74	74.11	74.58	72.22
No	71.00	62.53	59.63	74.25
**P-value**	0.86	0.24	0.11	0.82
**Husband support**				
Yes	0.153	89.88	94.48	67.50
No	0.153	64.86	62.83	74.70
**P-value**	0.07	**0.001**	**0.001**	0.29
**Other support**				
Yes	83.31	68.62	65.02	67.29
No	70.12	73.36	74.15	73.65
**P-value**	0.14	0.59	0.28	0.43

Significant p-value at $p < 0.05$ (2 tailed).

Table S5: EORTC QLQ-C 23 Symptom scales and socio-demographic, clinical and social support variables (N=144)

**Variables**	**Systemic therapy side effect**	**Breast symptom**	**Arm symptoms**	**Upset by hair loss**
	Mean rank	Mean rank	Mean rank	Mean rank
**Residence**				
Refugee camp	93.73	94.05	88.59	69.32
Village	71.92	69.69	69.26	73.59
City	68.29	72.87	75.16	71.03
**P-value**	0.18	0.18	0.30	0.85
**Age in years**				
≤ 40	60.13	68.67	68.93	72.74
41–54	85.94	76.67	76.38	72.24
**P-value**	**0.000**	0.24	0.27	0.92
**Marital status**				
Unmarried	81.10	73.08	71.78	79.40
Married	71.11	72.41	72.62	71.39
**P-value**	0.31	0.94	0.93	0.29
**Education**				
Primary and less	94.92	93.53	94.13	76.71
Secondary	71.45	68.28	72.62	66.76
Colleague/University	67.05	70.19	66.26	76.28
**P-value**	**0.035**	0.058	**0.034**	0.20
**Working status**				
Employee	68.15	69.56	67.07	69.26
Housewife	74.23	73.67	74.66	73.79
**P-value**	0.42	0.59	0.32	0.43
**Number of children**				
≤5 persons	71.05	69.23	69.46	71.83
>5 persons	75.59	79.47	78.98	73.93
**P-value**	0.54	0.16	0.19	0.70
**Family size**				
≤5 persons	69.23	65.00	70.11	70.07
>5 persons	74.30	76.61	73.81	73.83
**P-value**	0.48	0.10	0.60	0.49
**Income (US $)**				
< 570	72.84	70.63	67.02	71.38
570-1140	73.18	68.76	71.81	75.45
> 1140	70.67	82.55	82.70	68.78
**P-value**	0.96	0.29	0.23	0.59
**Stage at diagnosis**				
Stage 1	76.59	55.91	60.77	72.50
Stage 2	74.73	79.02	80.84	77.55
Stage 3	71.09	69.20	67.26	67.32
Stage 4	64.20	74.40	71.10	76.90
**P-value**	0.86	0.30	0.23	0.33
**Chemotherapy**				
Yes	73.14	72.74	72.42	72.58
No	50.25	64.00	75.38	69.75
**P-value**	0.27	0.67	0.88	0.86
Radiotherapy				
Yes	74.45	73.75	77.07	71.73
No	67.24	69.14	60.21	74.58
**P-value**	0.35	0.55	**0.02**	0.63
**Hormonal therapy**				
Yes	70.94	72.20	73.04	68.91
No	81.64	74.26	69.33	93.55
**P-value**	0.27	0.83	0.70	**0.001**
**Biological treatment**				
Yes	67.41	67.61	67.34	71.39
No	77.88	77.67	77.96	73.67
**P-value**	0.13	0.14	0.12	0.66
**Surgical intervention**				
Complete Mastectomy	75.59	65.82	57.09	69.57
Partial Mastectomy	70.41	77.01	82.90	74.48
**P-value**	0.46	0.11	**0.001**	0.36

**Chronic disease**				
Yes	84.27	77.33	79.01	65.01
No	66.80	70.16	69.35	76.13
**P-value**	**0.01**	0.33	0.18	**0.04**
**Pain Medication**				
Yes	77.38	76.12	72.62	74.54
No	65.47	67.29	72.32	69.57
**P-value**	0.09	0.20	0.96	0.35
**Relatives cancer**				
Breast Cancer	73.19	66.41	72.35	83.85
Other Cancers	76.57	76.96	75.41	70.79
No history of cancer	70.04	73.77	71.11	66.79
**P-value**	0.74	0.51	0.88	**0.02**
**Family support**				
Yes	72.71	75.92	73.92	71.70
No	71.20	51.33	63.68	77.45
**P-value**	0.88	**0.01**	0.30	0.45
**Husband support**				
Yes	65.55	72.02	80.31	70.11
No	75.56	72.71	69.07	73.55
**P-value**	0.18	0.92	0.13	0.54
**Other support**				
Yes	69.13	64.85	66.87	70.31
No	73.24	74.19	73.74	72.98
**P-value**	0.64	0.29	0.44	0.69

Significant p-value at p < 0.05 (2 tailed).

Table S6: Doctor Satisfaction scales and socio-demographic, clinical and social support variables (N=144)

**Variables**	**Technical skills**	**Interpersonal skills**	**Information provision**	**Availability**
	Mean rank	Mean rank	Mean rank	Mean rank
**Residence**				
Refugee camp	69.23	78.23	68.86	55.36
Village	72.00	69.86	71.46	72.31
City	74.38	76.57	75.60	77.28
**P-value**	0.91	0.60	0.81	0.27
**Age in years**				
≤ 40	67.23	67.41	67.13	65.89
41–54	78.23	78.03	78.34	79.69
**P-value**	0.10	0.12	0.09	**0.04**
**Marital status**				
Unmarried	79.98	89.20	80.65	82.33
Married	71.29	69.81	71.19	70.92
**P-value**	0.38	**0.04**	0.32	0.24
**Education**				
Primary and less	76.82	90.18	89.29	88.63
Secondary	75.54	68.51	66.59	67.38
Colleague/University	68.64	70.94	72.85	72.36
**P-value**	0.57	0.12	0.09	0.13
**Working status**				
Employee	70.15	71.84	72.66	70.21
Housewife	73.44	72.76	72.44	73.41
**P-value**	0.66	0.90	0.97	0.66
**Number of children**				
≤5 persons	71.13	72.06	71.70	71.58
>5 persons	75.41	73.45	74.20	74.46
**P-value**	0.56	0.85	0.72	0.68
**Family size**				
≤5 persons	78.23	82.74	80.71	80.81
>5 persons	69.36	66.89	68.00	67.94
**P-value**	0.21	**0.02**	0.06	0.06
**Income (US $)**				
< 570	69.12	69.00	66.18	71.29
570-1140	71.64	71.41	76.88	74.05
> 1140	79.61	80.23	74.55	71.56
**P-value**	0.51	0.45	0.34	0.92
**Stage at diagnosis**				
Stage 1	75.77	75.64	79.86	80.05
Stage 2	80.14	77.43	78.24	77.11
Stage 3	65.57	67.78	68.27	67.49
Stage 4	69.65	71.15	58.60	70.00
**P-value**	0.26	0.62	0.31	0.53
**Chemotherapy**				
Yes	73.68	73.75	73.50	73.50
No	31.25	28.63	37.38	37.38
**P-value**	**0.04**	**0.03**	0.07	0.22
**Radiotherapy**				
Yes	71.00	71.18	72.87	71.57
No	76.54	76.06	71.51	75.00
**P-value**	0.472	0.526	0.856	0.650
**Hormonal therapy**				
Yes	71.84	72.50	72.72	71.28
No	76.36	72.52	71.24	79.67
**P-value**	0.64	0.99	0.87	0.37
**Biological treatment**				
Yes	75.99	72.01	71.11	71.94
No	68.81	73.01	73.96	73.09
**P-value**	0.29	0.88	0.66	0.86
**Surgical intervention**				
Complete Mastectomy	70.07	70.39	72.91	72.65
Partial Mastectomy	74.14	73.92	72.22	72.40

**P-value**	0.56	0.61	0.91	0.97
**Chronic disease**				
Yes	74.07	79.77	78.79	77.80
No	71.74	68.98	69.45	69.93
**P-value**	0.74	0.13	0.18	0.27
**Pain Medication**				
Yes	74.87	75.36	73.59	74.62
No	69.08	68.38	70.93	69.45
**P-value**	0.40	0.31	0.69	0.44
**Relatives cancer**				
Breast Cancer	75.95	70.45	69.84	72.80
Other Cancers	65.51	64.74	69.36	67.50
No history of cancer	74.04	77.62	75.64	74.86
**P-value**	0.49	0.29	0.66	0.67
**Family support**				
Yes	74.35	73.97	73.82	74.99
No	61.05	63.38	64.33	57.08
**P-value**	0.17	0.28	0.32	0.06
**Husband support**				
Yes	74.69	74.72	76.07	76.07
No	71.54	71.53	70.93	70.93
**P-value**	0.67	0.66	0.47	0.96
**Other support**				
Yes	65.19	67.15	69.87	65.35
No	74.11	73.68	73.08	74.08
**P-value**	0.31	0.46	0.70	0.31

Significant p-value at $p < 0.05$ (2 tailed).

Table S7: Nurse Satisfaction scales and socio-demographic, clinical and social support variables (N=144)

**Variables**	**Technical skills**	**Interpersonal skills**	**Information provision**	**Availability**
	Mean rank	Mean rank	Mean rank	Mean rank
**Residence**				
Refugee camp	76.23	75.55	79.86	68.59
Village	72.22	73.01	71.90	71.86
City	72.14	70.66	71.87	74.85
**P-value**	0.95	0.92	0.81	0.87
**Age in years**				
≤ 40	68.05	69.46	67.37	67.78
41–54	77.33	75.80	78.08	77.63
**P-value**	0.17	0.35	0.10	0.14
**Marital status**				
Unmarried	74.15	81.30	80.85	87.18
Married	72.23	71.08	71.15	70.13
**P-value**	0.84	0.30	0.31	0.07
**Education**				
Primary and less	78.50	86.74	84.89	89.82
Secondary	70.01	68.83	67.07	68.65
Colleague/University	72.96	71.64	73.69	70.93
**P-value**	0.72	0.25	0.22	0.12
**Working status**				
Employee	75.22	68.46	68.50	67.04
Housewife	71.42	74.11	74.09	74.67
**P-value**	0.61	0.45	0.44	0.30
**Number of children**				
≤5 persons	72.26	71.95	72.58	72.14
>5 persons	73.02	73.67	72.33	73.26
**P-value**	0.91	0.81	0.97	0.87
**Family size**				
≤5 persons	77.37	78.68	78.22	76.95
>5 persons	69.83	69.11	69.37	70.06
**P-value**	0.28	0.18	0.20	0.32
**Income (US $)**				
< 570	66.13	70.06	69.64	71.97
570-1140	76.80	73.76	74.71	73.03
> 1140	74.78	74.11	73.00	72.36
**P-value**	0.36	0.86	0.79	0.99
**Stage at diagnosis**				
Stage 1	79.77	76.14	71.14	82.32
Stage 2	81.38	79.37	81.81	80.03
Stage 3	64.55	68.22	67.25	67.32
Stage 4	64.65	56.45	54.15	51.70
**P-value**	0.11	0.27	0.09	0.09
**Chemotherapy**				
Yes	73.04	73.06	73.49	72.67
No	53.50	53.00	38.00	66.63
**P-value**	0.34	0.33	0.08	0.76
**Radiotherapy**				
Yes	73.25	73.25	71.92	73.35
No	70.49	70.49	74.06	70.21
**P-value**	0.71	0.72	0.77	0.67
**Hormonal therapy**				
Yes	72.53	73.28	73.17	73.15
No	72.33	67.93	68.57	68.71
**P-value**	0.98	0.58	0.62	0.64
**Biological treatment**				
Yes	72.95	71.47	69.09	68.86
No	72.03	73.59	76.10	76.34
**P-value**	0.89	0.75	0.29	0.26
**Surgical intervention**				

Complete Mastectomy	69.35	67.77	70.52	70.48
Partial Mastectomy	74.62	75.69	73.84	73.86
**P-value**	0.44	0.25	0.62	0.62
**Chronic disease**				
Yes	76.88	73.72	78.27	78.39
No	70.38	71.91	69.71	69.64
**P-value**	0.36	0.80	0.22	0.22
**Pain Medication**				
Yes	74.49	76.39	73.69	74.45
No	69.64	66.89	70.79	69.69
**P-value**	0.48	0.17	0.66	0.48
**Relatives cancer**				
Breast Cancer	71.19	72.39	70.06	68.85
Other Cancers	67.07	63.87	69.87	66.97
No history of cancer	76.01	76.94	75.25	77.42
**P-value**	0.55	0.31	0.73	0.36
**Family support**				
Yes	74.55	73.08	74.15	74.73
No	59.78	68.90	62.28	58.68
**P-value**	0.13	0.67	0.21	0.09
**Husband support**				
Yes	79.64	80.78	76.53	77.42
No	69.36	68.86	70.73	70.34
**P-value**	0.16	0.11	0.42	0.33
**Other support**				
Yes	64.90	72.46	65.67	67.04
No	74.17	72.51	74.00	73.70
**P-value**	0.29	0.99	0.33	0.44

Significant p-value at $p < 0.05$ (2 tailed).

Table S8: Others Satisfaction scales and socio-demographic, clinical and social support variables (N=144)

Variables	Exchange information	Kindness, helpfulness, information giving	Waiting Time	Access	Comfort/cleanliness	General satisfaction
	Mean rank	Mean rank	Mean rank	Mean rank	Mean rank	Mean rank
**Residence**						
Refugee camp	64.95	64.82	71.64	74.59	66.27	58.09
Village	73.93	74.58	75.03	74.64	76.56	74.83
City	71.44	70.10	67.43	67.48	65.60	71.31
**P-value**	0.74	0.67	0.57	0.61	0.27	0.40
**Age in years**						
≤ 40	65.82	66.81	70.35	70.53	73.75	67.65
41–54	79.76	78.68	74.84	74.64	71.14	77.77
**P-value**	**0.029**	0.07	0.49	0.53	0.69	0.12
**Marital status**						
Unmarried	81.23	77.08	72.10	68.95	61.73	78.25
Married	71.09	71.76	72.56	73.07	74.24	71.57
**P-value**	0.27	0.58	0.96	0.66	0.18	0.48
**Education**						
Primary and less	79.74	87.55	87.95	84.82	83.08	87.89
Secondary	73.42	69.26	68.97	68.68	72.56	66.73
Colleague/University	69.65	71.04	71.17	72.31	69.45	73.13
**P-value**	0.58	0.21	0.17	0.31	0.41	0.12
**Working status**						
Employee	69.88	69.06	65.01	70.13	64.15	73.02
Housewife	73.54	73.87	75.48	73.44	75.83	72.29
**P-value**	0.60	0.51	0.14	0.65	0.10	0.92
**Number of children**						
≤5 persons	70.81	74.15	73.11	73.11	70.78	71.52
>5 persons	76.11	68.99	71.21	71.21	76.16	74.59
**P-value**	0.43	0.47	0.78	0.37	0.44	0.66]
**Family size**						
≤5 persons	74.61	80.75	78.84	79.21	73.35	79.10
>5 persons	71.34	67.97	69.02	68.82	72.03	68.88
**P-value**	0.62	0.06	0.15	0.13	0.84	0.13
**Income (US $)**						
< 570	67.27	69.33	70.12	71.55	69.64	66.50
570-1140	78.56	73.88	73.68	71.87	73.98	75.64
> 1140	69.64	75.06	74.17	75.23	74.38	76.36
**P-value**	0.26	0.77	0.85	0.90	0.80	0.38
**Stage at diagnosis**						
Stage 1	80.64	63.09	61.68	57.14	54.18	71.77
Stage 2	75.42	81.35	78.68	84.65	79.05	79.03
Stage 3	70.15	68.75	70.26	65.58	71.08	68.11
Stage 4	61.90	55.90	63.10	63.90	63.85	63.95
**P-value**	0.60	0.13	0.38	**0.02**	0.21	0.41
**Chemotherapy**						
Yes	73.08	73.14	73.17	73.17	73.23	73.35
No	52.38	50.00	49.00	49.00	46.88	42.75
**P-value**	0.28	0.25	0.22	0.23	0.18	0.12
**Radiotherapy**						
Yes	71.29	74.33	75.50	73.90	74.10	71.73
No	75.77	67.56	64.42	68.74	68.19	74.56
**P-value**	0.53	0.37	0.13	0.49	0.42	0.70
**Hormonal therapy**						
Yes	71.29	72.46	73.44	74.20	73.80	72.85
No	79.57	72.74	66.98	62.55	64.88	70.48
**P-value**	0.36	0.97	0.48	0.21	0.33	0.79
**Biological treatment**						
Yes	76.57	73.80	69.58	72.86	73.87	73.43
No	68.19	71.13	75.59	72.12	71.05	71.52
**P-value**	0.18	0.69	0.35	0.91	0.66	0.77
Surgical intervention						
Complete Mastectomy	74.15	71.13	69.33	64.36	73.64	71.30

Partial Mastectomy	71.39	73.42	74.64	77.99	71.73	73.31
**P-value**	0.67	0.73	0.42	**0.04**	0.77	0.76
**Chronic disease**						
Yes	78.53	80.70	79.81	74.44	70.04	76.98
No	69.58	68.53	68.96	71.56	73.69	70.33
**P-value**	0.18	0.09	0.12	0.68	0.60	0.34
**Pain Medication**						
Yes	73.95	72.75	73.57	72.79	69.99	74.19
No	70.42	72.14	70.96	72.08	76.12	70.06
**P-value**	0.58	0.92	0.69	0.91	0.35	0.53
**Relatives cancer**						
Breast Cancer	74.19	73.00	75.24	78.83	76.49	71.78
Other Cancers	77.04	66.54	67.24	60.66	62.91	69.74
No history of cancer	69.22	75.23	73.58	74.84	75.05	74.32
**P-value**	0.58	0.58	0.64	0.11	0.24	0.84
**Family support**						
Yes	74.46	73.04	75.16	73.89	74.45	73.94
No	60.38	69.15	56.00	63.90	60.43	63.58
**P-value**	0.12	0.68	**0.04**	0.29	0.13	0.27
**Husband support**						
Yes	72.80	80.48	80.80	80.67	80.92	79.09
No	72.37	68.99	68.85	68.91	68.80	69.60
**P-value**	0.95	0.11	0.09	0.10	0.08	0.18
**Other support**						
Yes	58.71	64.90	59.46	67.77	64.10	64.06
No	75.54	74.17	75.37	73.54	74.35	74.36
**P-value**	**0.041**	0.28	0.06	0.50	0.22	0.22

Significant p-value at $p < 0.05$ (2 tailed).
